# Cohort Profile: A Descriptive Analysis of Patients Aged 75 Years and Older with Public Health Coverage in Madrid at Baseline, Including a 5-Year Preobservational Period (2015–2019)

**DOI:** 10.3390/jcm15020571

**Published:** 2026-01-10

**Authors:** Victor Iriarte-Campo, Pilar Vich-Perez, José M. Mostaza, Carlos Lahoz, Juan Cárdenas-Valladolid, Paloma Gómez-Campelo, Belén Taulero-Escalera, F. Javier San-Andrés-Rebollo, Fernando Rodriguez-Artalejo, Enrique Carrillo-de Santa Pau, Lucía Carrasco, Miguel Angel Salinero-Fort

**Affiliations:** 1 Frailty, Multimorbidity Patterns and Mortality in the Elderly Population Residing in the Community—Hospital La Paz Institute for Health Research, IdiPAZ, 28046 Madrid, Spain; pilar.vich@salud.madrid.org (P.V.-P.); juan.cardenas@salud.madrid.org (J.C.-V.); pagocam@hotmail.com (P.G.-C.); belentauesc@gmail.com (B.T.-E.); fcojavier.san@salud.madrid.org (F.J.S.-A.-R.); 2 Los Alpes Health Centre, 28022 Madrid, Spain; 3 Lipid and Vascular Risk Unit, Department of Internal Medicine, Hospital Carlos III, 28029 Madrid, Spain; josemaria.mostaza@salud.madrid.org (J.M.M.); carlos.lahoz@salud.madrid.org (C.L.); 4 Faculty of Nursing, Alfonso X El Sabio University, 28691 Madrid, Spain; 5 La Paz University Hospital Biomedical Research Foundation, 28046 Madrid, Spain; 6 Foundation for Biomedical Research and Innovation in Primary Care (FIIBAP), 28003 Madrid, Spain; 7 Las Calesas Health Centre, 28026 Madrid, Spain; 8 Department of Preventive Medicine and Public Health, Universidad Autónoma de Madrid, 28029 Madrid, Spain; fernando.artalejo@uam.es; 9 Networked Biomedical Research Centre, Epidemiology and Public Health CIBERESP, 28229 Madrid, Spain; 10 Cardiovascular Nutritional Epidemiology, IMDEA Food, 28049 Madrid, Spain; 11 Computational Biology Group, IMDEA Food, 28049 Madrid, Spain; enrique.carrillo@imdea.org (E.C.-d.S.P.); lucia.carrasco@nutricion.imdea.org (L.C.); 12 Network for Research on Chronicity, Primary Care, and Health Promotion (RICAPPS), 28029 Madrid, Spain

**Keywords:** electronic health records, databases, factual, aged, 80 and over, cardiovascular diseases, chronic disease, risk factors, primary health care

## Abstract

**Background/Objectives**: Population aging increases the healthcare burden of chronic diseases. We aimed to characterize the sociodemographic and clinical characteristics of Aged Madrid, a cohort comprising 98.6% of the population aged 75 years and older in Madrid, Spain. **Methods**: Observational study with a five-year retrospective baseline period (2015–2019) to assess baseline vascular and metabolic risk. Data were taken from primary care electronic medical records, hospital discharge summaries, and pharmacy records. **Results**: 587,603 individuals (mean age: 84 years ± 5.8 years, 61.3% women) were analysed. Obesity affected 31.3% (more frequent in women), while type 2 diabetes occurred in 23.8% (predominantly in men). Hypertension (52.8%), dyslipidaemia (61.6%), and chronic kidney disease (21.7%) were more frequent in women. Atrial fibrillation was the leading cardiovascular condition in women (15.1%), while acute myocardial infarction predominated in men (8.2%). The most prescribed drug classes were antihypertensives (53.8%), statins (44.2%), and oral antidiabetics (26.4%). Among antihypertensives, diuretics (53.9%), ACE inhibitors (27.4%), and ARBs (25.3%) were most used, often in combinations such as diuretics + ACE inhibitors (30.1%). Diabetes treatments favoured metformin and DPP-4 inhibitors; 5.2% received insulin. **Conclusions**: Sex-based differences emerged in biochemical, anthropometric, and lifestyle variables. Men showed a higher prevalence of cardiovascular diseases and several cardiometabolic risk factors, while women used fewer lipid-lowering and antidiabetic agents. Diuretics were the predominant antihypertensives, and antidiabetic therapy largely followed guideline recommendations. Although 60% of statin users had no prior cardiovascular disease, and their use was concentrated mainly among individuals with major cardiometabolic risk conditions and declined with advancing age, suggesting an age- and risk-sensitive prescribing pattern rather than indiscriminate use.

## 1. Introduction

Aging of the population of developed countries is increasing the healthcare burden and costs associated with chronic diseases, particularly cardiovascular risk comorbidities (CVRCs), such as arterial hypertension (HT) and obesity, whose incidence is increasing rapidly [1]. Additionally, owing to inherent pathophysiological processes and lower physical activity among elderly individuals, other CVRCs, such as type 2 diabetes mellitus (T2DM) and dyslipidaemia (DLD), are also highly prevalent [2,3,4,5,6,7]. Factors contributing to this increase in prevalence include the progressive improvement in the management of cardiovascular risk conditions and the gradual decrease in cardiovascular mortality in recent decades [8,9,10,11].

CVRCs predispose individuals to cardiovascular disease (CVD) and functional decline [12], and they are also leading causes of death [13]. Almost one-third of new ischemic strokes in Europe occur in patients aged 80 years or older [14]. According to the 2022 Annual Report of the Spanish National Health System [15], patients with CVD especially older men, tend to visit primary care more frequently. The prevalence of ischemic heart disease is greater among men aged ≥70 years, and CVD accounts for a significantly greater proportion of hospitalizations in men. In contrast, women have a lower incidence of CVD but a poorer prognosis and higher mortality rates [16]. Indeed, CVD is the leading cause of death in women, accounting for approximately one-third of female deaths in the USA [17].

Chronic HT, T2DM, obesity, smoking, and sleep disorders significantly contributes to cognitive decline and dementia in aging individuals [18]. Additionally, an unprecedented increase in the global incidence of cancer incidence is expected, since age is a risk factor for both cancer and diabetes [19] and T2DM and obesity are associated with an increased risk of colorectal and postmenopausal breast and endometrial cancer [19].

In older patients, the high burden of multimorbidity and CVD frequently leads to polypharmacy, commonly defined as the concurrent use of five or more medications [20]. Polypharmacy is highly prevalent in this population and has been linked to adverse outcomes, including hospitalization, functional decline, and mortality [21]. In this context, a detailed description of cardiovascular and cardiometabolic pharmacotherapy, including patterns of multiple drug use such as treatment with three or more antihypertensive agents, is essential if we are to understand the medication burden of and potential risks faced by older people in routine clinical practice.

Finally, although overall mortality rates declined significantly by the late twentieth century, diseases such as cancer, T2DM, respiratory disease and CVDs accounted for nearly 70% of deaths worldwide [1], with mortality rates from Alzheimer’s disease, heart disease, cerebrovascular disease, influenza, and pneumonia increasing exponentially with age [22]. Nevertheless, elderly patients are often underrepresented in studies of chronic diseases [23].

Population-based cohorts generated from electronic medical records (EMRs) [1] for prospective studies with a run-in period [24] provide valuable insights into aging and chronic conditions, particularly regarding sex differences, and are well-suited for exploring the sociodemographic and clinical characteristics of older adults.

The life expectancy of the population in the region of Madrid is 85.2 years, the highest in the European Union (EU) and four years higher than the average of all member states. In addition, the European Commission’s Innovation Partnership for Active and Healthy Ageing awarded Madrid the status of a four-star reference site for 2019 and 2022 [25]. Therefore, Madrid represents an appropriate setting for deploying measures to deepen knowledge of the aging process in persons who maintain a good quality of life. In addition, life expectancy without disability in Madrid is among the highest in EU regions.

Stratified by age and sex, the present analysis describes the Aged-Madrid cohort in terms of sociodemographic distribution, comorbidities, risk factors, and treatment patterns for CVRCs in order to propose strategies aimed at improving cardiovascular risk control. We also describe changes between the yearly mean values of the first and last available years of anthropometric and biochemical variables during the retrospective baseline period (2015–2019), stratified by sex and age group.

## 2. Materials and Methods

### 2.1. Study Design and Participants

Aged-Madrid is a population-based cohort of individuals aged 75 years and older from Spain, with baseline data collected over a 5-year retrospective baseline period (2015–2019). We used EMRs to perform a population-based study of the Aged-Madrid cohort, which includes all individuals aged 75 years or older who were alive and residing in the Community of Madrid on 1 January 2020, the predefined key date. These criteria were met by 587,603 participants, all of whom were covered by the Spanish National Health System, a universal, publicly funded service.

During the first data extraction, a technical error temporarily included 5746 individuals who had died before the index date, resulting in an initial dataset of 593,392. This issue was corrected, ensuring that inclusion was based solely on age, residence, and vital status on 1 January 2020, without taking into account survival in previous years.

According to the Spanish National Statistics Institute [26], our study participants represented 98.6% of the total population (595,652 individuals) of those aged 75 and older in the Madrid region in 2020.

**Figure 1:**
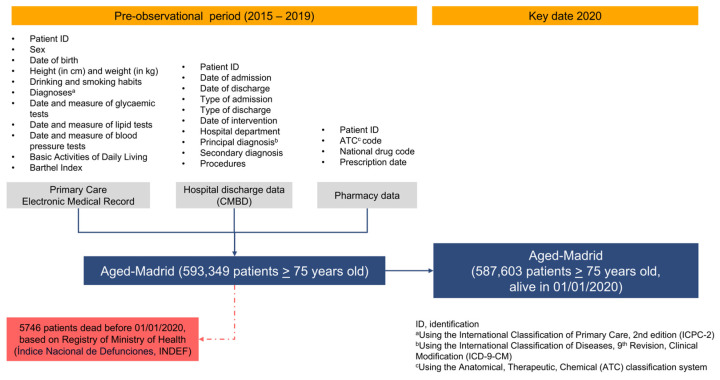
Flow chart of patients from original Aged-Madrid cohort..

The flow chart of individuals participating in the Aged-Madrid cohort is shown in Figure 1. The original variables available in the Aged-Madrid Database are detailed by data source.

### 2.2. Data Source

The variables and data analysed were obtained from several sources. The first was primary care EMRs, which have been validated for their main diagnoses (T2DM, HT, atrial fibrillation [AF], acute myocardial infarction [AMI], and stroke) [27,28,29] and are habitually used in epidemiologic studies [30]. The second was hospital discharge data Spanish Minimum Basic Dataset of Hospital Discharges [CMBD]). Mortality data were obtained from the National Death Index of the Ministry of Health (INDEF) (Figure 1).

Diagnoses were confirmed using hospital discharge records as the gold standard for major conditions such as AMI, stroke, and heart failure (HF). These were prioritized in the case of discrepancies. Nevertheless, primary care diagnoses of AMI, stroke, AF, diabetes, and HT were also included, as supported by recent validation studies in EMRs conducted by our group. For lifestyle-related variables (physical activity, sedentary behavior, smoking, and alcohol consumption), we prioritized primary care records, as these are routinely collected in local health centers by nurses specialized in community care and disease prevention.

### 2.3. Study Variables

Body mass index (BMI) was expressed as kg/m^2^ (BMI = weight/height^2^) and categorized as underweight/normal weight if BMI < 25, overweight if BMI = 25–29, and obesity if BMI ≥ 30 [31].

Blood from the patients at each health centre was sent to its reference laboratory in tubes without anticoagulants. Next, the blood was centrifuged at 3000× *g* for 30 min to obtain serum for biochemical analyses. The samples were then stored at 4–6 °C in a dark environment. All blood samples were processed in the laboratory within 12 h of collection. We also used the following levels: fasting plasma glucose (FPG) in mg/dL, and HbA1c in %, plasma cholesterol and triglycerides in mg/dL, albuminuria in g/dL, albumin/creatinine ratio in mg/g, microalbuminuria in μg/mL, and creatinine in mg/dL. Systolic and diastolic blood pressure (SBP and DBP) were measured in mmHg using the standard procedure.

Socioeconomic deprivation was assessed using a continuous deprivation index [32]. The index was initially treated as a continuous variable. For analytical and descriptive purposes, it was categorized according to its empirical distribution. Participants in the lowest two tertiles (≤66 rd percentile) were classified as less disadvantaged, whereas those in the upper tertile (>66 rd percentile) were classified as more disadvantaged. This categorization was used to examine differences in lifestyle factors, anthropometric status, and cardiometabolic and cardiovascular comorbidities across socioeconomic strata.

According to the criteria of the International Diabetes Federation, pre-T2DM was defined as FPG ≥100 mg/dL–<126 mg/dL among those without T2DM diagnosis or not being treated with antidiabetics. The diagnoses of CVRCs and CVD were extracted from the primary care EMRs of the study participants.

Composite of cardiovascular endpoints (CCDEs) were defined to capture major vascular and cardiac outcomes. CCDE3 included AMI, stroke, or peripheral artery disease (PAD), while CCDE4 also incorporated HF. The time window for these composites was the lifetime prior to 2020, ensuring consistency across all participants.

Additionally, the estimated glomerular filtration rate (e-GFR) was calculated using the Chronic Kidney Disease Epidemiology Collaboration (CKD-EPI) method [33]. Patients with at least two e-GFR measurements taken over a follow-up period of more than three months were considered to have chronic kidney disease (CKD) if the average e-GFR was ≤0 mL/min/1.73 m^2^.

Changes in anthropometric and biochemical variables during the 2015–2019 retrospective baseline period were assessed by comparing the yearly mean values of the first and last available years among individuals with at least two observations, stratified by sex and age group. Paired t-tests were used to evaluate within-group changes. This descriptive approach was chosen to characterize overall population trends rather than individual-level longitudinal variability.

### 2.4. Data Processing and Analysis

Data from multiple sources were consolidated into a single anonymized database by external information system experts. To ensure plausibility, predefined thresholds were set a priori for each variable: SBP (<70 or >250 mmHg), DBP (<40 or >150 mmHg), FPG (<40 or >600 mg/dL), HbA1c (<3% or >15%), serum creatinine (>10 mg/dL), and triglycerides (>1000 mg/dL). Implausible values were corrected by cross-checking repeated measures within the same patient record; when correction was not possible, the data point was excluded. Duplicate records were removed only when the date of birth, sex, and multiple identical laboratory and blood pressure results coincided, thereby minimizing the risk of erroneously merging distinct individuals. Out-of-range values and duplicate participants were identified and managed using the Alteryx Designer x 64, version 2025.1.2.79 (Alteryx, Inc., Irvine, CA, USA) [34]. Finally, the curated dataset were analyzed using R software (version 4.2.2) [35].

Out-of-range thresholds were defined a priori for each variable to ensure plausibility as follows: SBP < 70 or >250 mmHg, DBP < 40 or >150 mmHg, FPG < 40 or >600 mg/dL, HbA1c < 3% or >15%, serum creatinine > 10 mg/dL, and triglycerides > 1000 mg/dL. Implausible values were corrected by cross-checking repeated measures within the same patient record; when no correction was possible, the data point was excluded. Duplicate removal was restricted to cases in which date of birth, sex, and multiple identical laboratory and blood pressure records coincided, thus minimizing the risk of erroneously merging distinct individuals.

The *t* test was used for hypothesis testing of continuous variables by sex, whereas analysis by age group was performed via the one-way ANOVA test. The Pearson’s chi-square test was used to assess differences between categorical variables.

To better understand the magnitude and differences between sex and age groups (75–80 years old vs. >90 years old), standard mean differences (SMDs) were calculated for baseline analytical parameters associated with cardiovascular risk.

Cramer’s V was used to assess the strength of association between categorical variables; values below 0.1 were considered to indicate very weak associations, even in large samples.

Given the large volume of patients in our database, and because small differences are likely to reach statistical significance in hypothesis testing, the strength of the associations between the treatments and the variables sex and age was determined using Cramer’s V. This standardizes the χ^2^ test to a range between 0 and 1, where <0.3 indicates a weak association [36].

Missing data were assessed using pattern analysis and variable-specific missingness rates. Missingness was concentrated in functional status (Barthel index), consistent with non-systematic recording in routine practice, and in some longitudinal laboratory measures reflecting incomplete annual testing. Given the descriptive objective of the study and the structured nature of missingness, no multiple imputations were performed. Analyses were conducted using available data, and denominators are reported for each variable to reflect variable-specific completeness.

The distribution of missing values across the study variables is provided in Supplementary Table S1. Supplementary Figure S1 presents a heatmap illustrating the presence and absence of data across study variables, while the pattern of missing values is shown in Supplementary Figure S2.

## 3. Results

### 3.1. Baseline Characteristics and Comorbidities

After excluding 5746 patients who died before 1 January 2020 (Figure 1), the Aged-Madrid cohort consisted of 587,603 individuals with a mean age of 83.5 years (standard deviation, [SD], 5.8). Table 1 shows that all comparisons were significant in both sex and age group analyses, with *p* < 0.001. Women accounted for 61.7% of the total population. Of these, 35.6% were aged between 75–80 years, and 15.4% were aged over 90 years. Among the males, 43.6% were aged between 75 and 80 years, and 9.4% were over 90 years. For the 81–85- and 86–90-year-old age groups, the percentages of women and men were very similar, (approximately 27% and 21%, respectively) (Table 1). As a result, the proportion of patients diagnosed with Alzheimer’s disease was greater among women than among men (9.1% vs. 5.9%), and three times more prevalent among patients aged >90 years (15.8%) than among those aged 75–80 years (3.3%) (Supplementary Table S2).

Alcohol consumption and smoking were more prevalent among men than women (2.6% and 8.3% among men and 0.3% and 2.7% among women, respectively) (Table 1).

With respect to weight, 44.8% of the participants with BMI available (n = 423,021) were overweight, (40.9% of women and 50.9% of men). Additionally, 31.3% of the participants were obese (33.7% women and 27.5% men). <25% of both sexes was underweight or normal weight.

**Figure 2:**
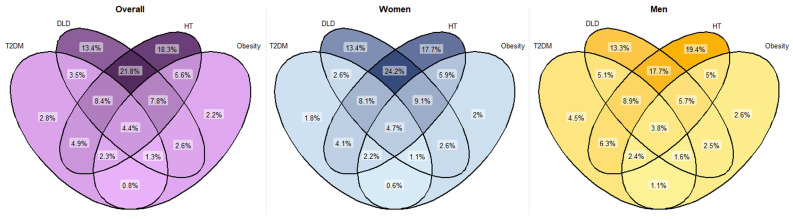
Venn diagram of prevalence of T2DM, DLD, HT and obesity comorbidities. Percentages calculated based on the total population and on the population by sex. χ2 tests were used to compare categorical variables. All comparisons were significant (p < 0.001). DLD: dyslipidaemia; HT: hypertension; T2DM: type 2 diabetes mellitus.

The most common weight category for each age group was overweight. The balance between underweight/normal weight and obesity differed according to age group. Among those aged 75–80 years, more people were obese (34.2%) than under/normal weight (21.0%). In the age group over 90 years, these proportions were reversed (22.5% vs. 34.2%) (Table 1). 2150 subjects had type 1 diabetes mellitus and 76,216 people met the prediabetes criteria (see Supplementary Table S2). The prevalence of T2DM was 23.8%, with more men than women (27.3% and 21.5%, respectively). However, the prevalence of HT and DLD was higher in women (65.0% vs. 56.0% and 56.2% vs. 47.4%, respectively). The prevalence of HT increased progressively with increasing age. In contrast, the prevalence of DLD remained stable in patients aged 86–90 years, with a decrease in the group aged >90 years (Table 1). Both conditions were more prevalent among women in all the age groups. Joint analysis of men and women revealed the higher prevalence of DLD + HT among women (24.2% vs. 17.7%) to be significant (p < 0.001) (see Figure 2), with age group-specific rates being comparable to those observed in the general population.

CKD was present in 21.7% of the population for whom the e-GFR was available (n = 385,062), with a higher prevalence noted among women (22.4%) than men (20.6%). Furthermore, the prevalence of CKD increased with each age group (Table 1).

The three most common CVDs were AF (15.1% of patients), HF (6.3%) and stroke (6.1%). Apart from HF, these conditions were more common in men and tended to increase with patient age. The most common CVD in men was AMI, occurring in 8.2% (Table 1).

Finally, both CCDE3 and CCDE4 were clearly more prevalent in men, with a more pronounced and sustained age-related increase for CCDE4. For further details on these and other conditions, refer to Supplementary Table S2. Given the low percentage (34.19%) of available values for the measurement of the study participants’ ability to perform basic activities of daily living (Barthel index), the results for this variable are also shown in Supplementary Table S2.

### 3.2. Baseline Characteristics and Comorbidities Stratified by Socioeconomic Deprivation Status

Stratifying by the deprivation index (patients with more disadvantages vs. patients with fewer disadvantages), the difference in average age was less than one year (*p* < 0.001). Otherwise, differences in the prevalence of obesity (35.5% vs. 29.0%), T2DM (27.1% vs. 22.1%), DLD (55.3% vs. 51.6%), HT (64.4% vs. 60.2%) and CKD (24.5% vs. 20.1%) were statistically significant (*p* < 0.001). Meanwhile, differences in the prevalence of CVD (AF, HF, stroke and AMI), although significantly higher among patients with more disadvantages, did not reach 1%, as was also the case with smoking and alcohol consumption. (Supplementary Table S3).

### 3.3. Baseline Analytical and Blood Pressure Parameters by Condition and Treatment

When analysed by condition, blood glucose and lipid levels were higher among treated patients than among untreated patients (a difference of 0.6% in HbA1c among patients diagnosed with T2DM, 22 mg/dL in LDL-cholesterol, and 6 mg/dL in triglycerides). No differences in HbA1c levels by sex or age group were observed, either in treated or untreated patients. Women had higher levels of both HDL-cholesterol and LDL-cholesterol than men, among patients treated for DLD and those who were untreated (*p* < 0.001). In contrast, sex differences in plasma triglyceride levels were only observed among treated patients (Table 2).

Results for other baseline analytical parameters associated with cardiovascular risk are shown in Supplementary Table S4.

### 3.4. Baseline Treatments

In terms of treatment, around a quarter of patients were receiving some form of oral antidiabetic treatment (22.9% of women vs. 32.1% of men, *p* < 0.001). The most common were metformin (with nearly 5% more men than women) and dipeptidyl peptidase-4 (DPP-4) inhibitors. Moreover, 5.2% of patients were being treated with insulin (5.7% of women vs. 4.9% of men, *p* < 0.001), with a stable prescription trend across age groups (Table 3). Very low frequencies were recorded for treatment with thiazolidinediones (0,1%), with sodium-glucose co-transporter 2 inhibitors, (SGLT2i) (1.5%), and glucagon-like peptide-1 receptor agonists (GLP-1 RA) (0.5%) (see Supplementary Table S5).

The most frequently prescribed drugs were antihypertensives, including diuretics (53.9% overall; 57.9% of women vs. 47.4% of men, *p* < 0.001), and renin‒angiotensin‒aldosterone system (RAAS) blockers (52.7% of patients, with similar proportions for women and men, *p* < 0.001). By age group, the greatest difference in antihypertensive prescriptions was observed for diuretics (41.4% in the population aged 75–80 years vs. 70.8% in those over 90 years) (Table 3).

Additionally, 44.2% of the population (42.2% of women vs. 47.3% of men; *p* < 0.001) were taking statins, with a slight decrease in use with age. This was particularly notable in those aged over 90 years (Table 3). In 60.0% of cases, patients treated with statins had no history of CVD, defined as the presence of AMI, stroke, PAD, HF, angina, chronic ischemic heart disease, transient ischemic attack, or AF, and 40% were in secondary prevention.

The strength of the association between sex and age was weak for all the variables (Cramer’s V ≤ 0.1) (Table 3).

For further information on other baseline treatments, see Supplementary Table S5.

**Figure 3:**
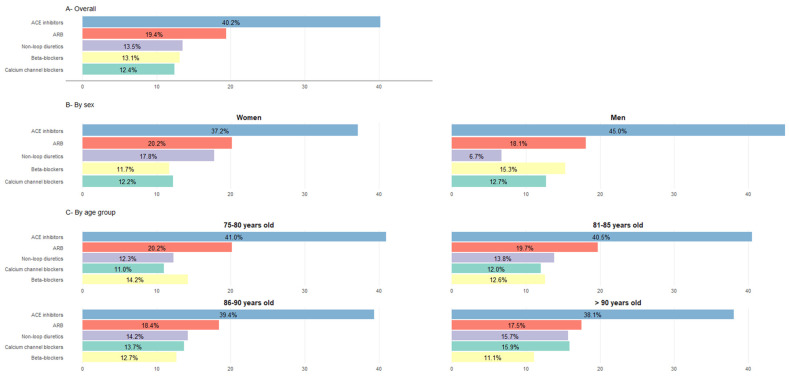
Use of antihypertensive drugs as monotherapy (in% patients). Percentages calculated based on the total population treated with a monotherapy regimen within each group. χ^2^ tests were used to compare categorical variables. All comparisons by sex and by age group were significant (*p* < 0.001). Categories of antihypertensive drugs not represented: alpha-blockers used in ≤1% of treated patients across all groups, except among men (1.7%); aldosterone antagonists used in <1% % of treated patients in all groups. ACE inhibitors: angiotensin-converting enzyme inhibitors; ARB: angiotensin II receptor blockers.

A total of 21.3% of patients treated with antihypertensive drugs were on monotherapy, 40.2% of them with angiotensin-converting enzyme (ACE) inhibitors and 19.4% with angiotensin II receptor blockers (ARBs). See Figure 3 for further information on the distribution of antihypertensive monotherapy regimens by sex and age group in the general population.

**Figure 4:**
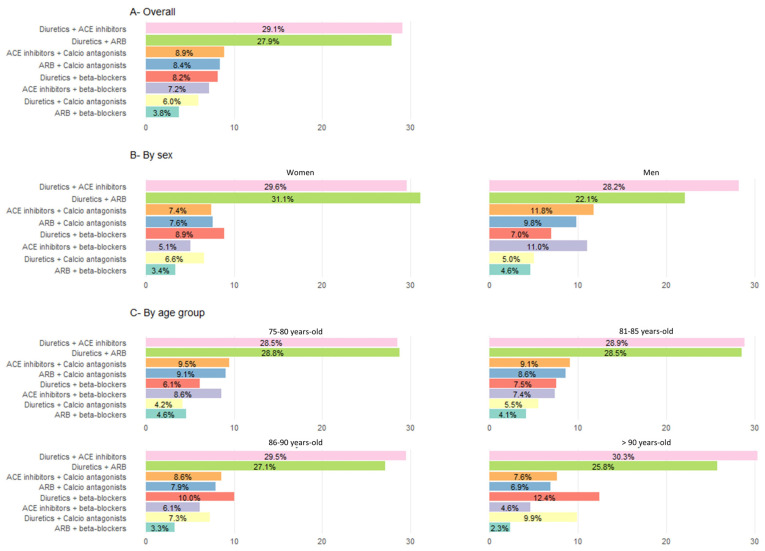
Use of antihypertensive drugs as dual therapy (in% patients).Percentages calculated based on the total population treated with a monotherapy regimen within each group. χ^2^ tests were used to compare categorical variables. All comparisons by sex and by age group were significant (*p* < 0.001). Categories of antihypertensive drugs not represented: ACE inhibitors + ARB in ≤0.5% of treated patients across all groups. ACE inhibitors: angiotensin-converting enzyme inhibitors; ARB: angiotensin II receptor blockers.

Additionally, 21.8% of patients were treated with two drugs. The main dual combinations were diuretics and angiotensin-converting enzyme inhibitors (ACE inhibitors) (29.1%), and diuretics and ARBs (27.9%) (see Figure 4 for more information, including distribution by sex and age group). Finally, 25.9% of patients were treated with three or more drugs, while 31.0% of patients did not receive antihypertensive treatment.

Analysis of these data by sex, revealed that although all differences were statistically significant, the most pronounced disparities between men and women were among those treated with non-loop diuretics (41.2% women vs. 33.0% men) and those treated with ACE inhibitors (25.7% women vs. 30.1% men). By age group, the only segment with a rising prescription pattern was patients treated with diuretics (Table 3) while a marked decrease was observed in the use of RAAS blockers.

### 3.5. Variation Within the Retrospective Baseline Period (2015–2019)

The time range between the first and last measurements of BMI, FPG, LDL-cholesterol, and SBP/DBP in the study patients was 3.5–4.3 years, with very slight variations observed in each variable. Changes in BMI, FPG, LDL-cholesterol, SBP, and DBP during the 2015–2019 baseline period, stratified by sex and age group, are presented in Supplementary Tables S6–S10.

### 3.6. Statins in Patients Without Cardiovascular Disease

Among patients without prior CVD, statin use varied substantially according to the presence of major cardiometabolic risk conditions (Table 4). Among individuals with T2DM, 56.0% were receiving statins, with higher proportions observed in women (57.1%) and in those aged 75–80 years (62.4%), followed by a progressive decline with advancing age. Similarly, among patients with DLD, 60.9% received statin therapy, with a higher prevalence in men (63.3%) and in younger age groups within the cohort, decreasing to 41.0% among those aged ≥90 years.

In contrast, statin use was less frequent among patients with HT (42.8%) and CKD (44.4%) in the absence of established CVD. In both conditions, statin treatment was more common in individuals aged 75–80 years and declined markedly in the oldest age groups, reaching 24.3% among hypertensive patients and 25.9% among those with CKD aged ≥90 years.

Overall, these findings indicate that a substantial proportion of statin use in patients without prior CVD was in the context of high-risk cardiometabolic conditions, with treatment patterns strongly influenced by age (Table 4).

## 4. Discussion

This study presents the design and findings regarding the sociodemographic and clinical characteristics of patients aged 75 years and older from the Aged-Madrid baseline cohort upon completion of the retrospective baseline period (2015–2019). The main diagnoses in the EMRs (T2DM, HT, AF, AMI, and stroke) in this study have been previously validated by our group [27,28,29]. Given the large sample size, even for very small differences in the study variables between sexes or age groups were statistical significance. Therefore, the text primarily highlights differences that may have clinical or public health relevance.

In addition to the overall description of the findings, this study includes a description of anthropometric and biochemical variables during 2015–2019, aiming to highlight general trends within a large, population-based cohort with universal and free healthcare coverage. This design underscores the expectation that such a well-monitored population, with progressively increasing levels of clinical control during 2015–2019, will experience a lower incidence of future cardiovascular events than cohorts lacking comprehensive healthcare access. By focusing on descriptive yearly means rather on individual-level longitudinal models, our intention was to characterize the overall evolution of risk factors in this unique population context, thereby providing a benchmark for future comparative studies.

During the retrospective baseline period, we observed an overall improvement in anthropometric and biochemical values. Although the present study was descriptive and not intended to analyse the determinants of these changes, this trend possibly reflects better achievement of control targets, particularly for HT and diabetes, in line with increasingly strict recommendations in clinical practice guidelines. A detailed analysis of the factors driving this improvement should be addressed in future works

Socioeconomic deprivation emerged as a determinant of health status in the Aged-Madrid cohort. Individuals classified as more disadvantaged consistently exhibited a higher burden of cardiometabolic risk factors including diabetes, obesity, HT, CKD, and, to a lesser extent, established CVD. These findings align with extensive evidence linking socioeconomic disadvantages to cumulative exposure to adverse risk factors across the life course [37]. In very old populations, such patterns must also be interpreted in the context of selective survival and cohort effects, whereby individuals from more advantaged backgrounds may be more likely to survive into the oldest age groups [38]. However, this has not been the case in our study, where the difference found in the average age of patients was low. Nevertheless, the persistence of clear socioeconomic gradients in health among adults aged ≥75 years highlights the relevance of social determinants of health even at advanced ages and underscores their importance for both clinical risk stratification and public health planning.

Compared with the 2020 European Health Survey for Spain, which was conducted in patients aged 65 and older, our cohort had slightly lower proportion of overweight patients (44.8% vs. 46.19%) while the proportion of obese patients was significantly higher (31.3% vs. 21.2%) [39]. As in our study, the proportion of men was higher than that of women among overweight patients, whereas the opposite was observed among obese patients.

The prevalence of T2DM (23.8%) and its predominance in men were similar in both our study and the European Health Survey. In contrast, a Spanish study by Soriguer et al. reported a higher prevalence of T2DM in women (23.2% compared with 20.7%) and in patients aged 61–75 years (24.8% compared with 20.7% among those older than 76 years) [40].

In the case of HT, our prevalence of 61.6% falls between the 72.8% reported for those aged 80 years and over by Aguado et al. in 2009 [41] and the European Health Survey results of 55.2% for those aged 75–84 years and 59.2% for those aged 85 years and over. The discrepancies in prevalence rates may be due to differences in data collection methods, differences in the age groups studied, and potential recall bias, often leading to under-reporting cases [42]. In all these scenarios, a higher prevalence of HT was observed in women.

When our prevalence for type 2 diabetes, obesity, DLD, and HT were compared with those reported by Spijker et al. in 2023 (data for 2006–2017 from the Spanish National Health Survey [ENSE] for individuals aged 60–89 years), our percentages were higher in all cases (23.8% vs. 20.5%, 31.3% vs. 23.8%, 52.8% vs. 38.5%, and 61.6% vs. 47.7%, respectively), likely owing differences in the age range of the study populations and potential recall bias [43].

Comparison of our results with those of the EPICARDIAN study [44] showed that the percentages of patients with hypercholesterolemia in 2004 were higher than our DLD rates, both in patients aged 75–84 years (60.7% vs. approximately 55%) and in those aged 85 years or older (52.2% vs. 52.4% for ages 86–90 years and 43.9% for those over 90 years. This may be explained by improvements in disease control, including obesity, sedentary behaviour, and dietary adherence, since then [8]. In line with our results, hypercholesterolaemia was significantly more common among women in all age groups.

We note that the higher cholesterol levels observed in untreated than in treated dyslipidaemia should not be taken only as evidence of appropriate treatment targeting. Such differences may also be influenced by how often cholesterol is measured, physician prescribing practices, and patient adherence. Our analysis was descriptive, intended to show population-level trends rather than to draw causal conclusions.

In our study, the overall prevalence of AF was 15.1%, with 13.8% in women and 17.3% in men (*p* < 0.001). These rates were comparable to those reported in the PYCAF study, which found rates of 15.4% overall, (14.3% in women and 17.0% in men; *p* = 0.068). The PYCAF study was conducted on 2461 individuals aged 65 and older in 128 health centres in Spain [45].

The proportion of patients with HF in our study was 6.3% overall (5.9% in men and 6.5% in women; *p* < 0.001), which is in line with the findings of a recent systematic review of the prevalence and incidence of HF [46] that identified four Spanish studies where prevalence ranged from 2.8% (based on EMRs) [47] to 16.1% [48] among adults aged over 70. This study was based on Framingham criteria in primary care, which were then validated by cardiology specialists using echocardiography. In contrast, other publications, such as the PYCAF study [45], reported an overall HF prevalence of 9.1%, with a higher proportion in men than in women (11.3% vs. 7.6%, *p* = 0.002). Similarly, studies such as those of Redfield et al. (2003) [49] and van Riet et al. (2016) [50] showed the prevalence of HF to range between 4.7% and 13%, with men exhibiting higher rates than women, a pattern that differs from our observation.

Other studies, such as the PRICE study (conducted in primary care centres in Spain), reported a prevalence of 16.1% in individuals aged over 75 years [48]. This discrepancy may be explained by the active search for symptoms and signs of HF, which is not routinely performed in usual clinical practice. Furthermore, the presence of low functional status, coexisting obesity, and chronic obstructive pulmonary disease may complicate the diagnosis of HF in elderly populations unless actively sought.

The overall prevalence of stroke in our study was 6.1%, (7.3% in men and 5.3% in women; *p* < 0.001), slightly higher than that reported in the 2020 European Health Survey for Spain (3–4% of women and 2–3% of men) [39]. These differences could be attributed to the younger age of the survey participants and potential recall bias. In addition, our results were similar to those of another national study conducted in the central region of the Iberian Peninsula, which reported an age-standardised stroke prevalence (adjusted to the European population) ranging from 4.3% in the 75 to 79-year age group to 6.9% in those aged 85 years and older [51]. Recent data from the USA estimate a prevalence of 7.7% in individuals aged over 65 years (self-reported data from a survey) [40].

Compared with survey data [52], our figures for angina pectoris were similar (4.6% vs. nearly 3%), with higher sex disparities (6.7% of men and 3.3% of women in our study vs. approximately 2.5% and 3%, respectively). The prevalence of AMI was comparable (4.6% vs. close to 3%), although our study revealed twice the proportion of affected individuals (2.3% vs. approximately 1.7% in women and 8.2% vs. 4.3% in men). Nonetheless, these figures remain lower than those reported by Salari et al. in their 2023 meta-analysis, which estimated the global prevalence of AMI to be 9.5% in individuals aged >60 years [53].

Our HbA1c levels (7.0% in treated T2DM patients and 6.4% in non treated T2DM patients) were in line with the ADA recommendation [54] of <7.5% as a reasonable HbA1c target in healthy patients (those with few coexisting chronic illnesses and intact cognitive and functional status, the predominant profile in our population). Among patients with DLD receiving active treatment, LDL-cholesterol levels were consistent with the most recent and more stringent recommendations for patients at moderate risk (105 mg/dL in women and 94.8 mg/dL in men) [55], whereas among untreated patients, our figures (123 mg/dL in the total population, 125.2 mg/dL in women and 117.3 mg/dL in men) exceeded the 2016 recommendations (115 mg/dL) for these same patients [56]. For its part, the 2018 ESC/ESH Guidelines for the Management of Arterial Hypertension [57] recommend a treatment target for SBP of 130–139 mmHg and DBP < 80 mmHg in treated adults aged 65 years and over. When analysed by age group or by sex, the SBP and DBP levels of the patients in our cohort were in line with these recommendations.

Although many of the comparisons of baseline treatments revealed statistically significant differences owing to the large sample size, the low values of Cramer’s V (<0.1) indicate that sex and age account for very little of the variability in treatment allocation. This highlights that statistical significance does not necessarily imply clinical relevance, and that treatment decisions are likely driven by factors other than demographic characteristics.

The treatment patterns identified for T2DM, prioritizing metformin and DPP4 inhibitors far ahead of GLP-1 RA and insulin, align with therapeutic management recommendations for elderly patients [58,59,60] and are similar (26.4% of patients treated with oral antidiabetics and 5.2% with insulin) to those reported in the previous referred PYCAF study (24.6% and 7.4%, respectively) [45]. In the 2013 Japanese Hospital Database, 31.9% of T2DM patients studied (age ≥ 70 years, 45.7%), were taking biguanides and 24.3% were taking DPP4 inhibitors [61]. The discrepancy between the number of patients diagnosed with type 2 diabetes mellitus (T2DM) (139,582) and those treated with oral hypoglycaemic agents (155,091) could be explained by several factors. First, the 76,216 individuals with prediabetes may have been taking metformin, as recommended by the 2019 ESC Guidelines on diabetes, prediabetes, and cardiovascular diseases in collaboration with the EASD, based on evidence that metformin significantly reduces the development of diabetes over a 15-year period [62]. Furthermore, metformin may also be prescribed to patients with prediabetes and CVD in patients (because of its cardioprotective effects by reducing inflammation and improving endothelial function) [62]. Second, SGLT2i are sometimes prescribed to patients without diabetes for specific medical conditions such as CKD and HF [62,63] owing to their effect on blood pressure.

In terms of antihypertensive medication use, 53.9% of the patients were treated with diuretics, 27.4% were treated with ACE inhibitors, and 25.3% were treated with both monotherapy and combination therapy. These findings are slightly lower than expected for therapy with diuretics considering that they are the treatment of choice for controlling SBP in elderly patients [64], although they are similar to those reported by Rodríguez-Roca et al. in their cross-sectional multicentre Spanish study of patients with HT aged at least 80 years in primary care treated with ACE inhibitors (30.7%) and ARBs (28.9%). However, in the case of diuretics, a significantly different percentage was recorded (21.4%) [65]. Considering that loop diuretics should be avoided in elderly patients [57], their use in 15.8% of our patients in our cohort may be justified by prescriptions for concomitant diseases. Among those receiving monotherapy, ACE inhibitors followed by ARBs were the most common therapeutic groups, both in the overall population and in the analyses stratified by age and sex. For combination therapy, with figures that should potentially reach more patients as in the present case, the percentages of patients treated with diuretics receiving ACE inhibitors (29.1% in our study vs. 34.9%), ACE inhibitors with calcium channel blockers (8.9% vs. 11.3%), and diuretics with beta-blockers (8.2% vs. 5.9%) were also very similar to those reported by Rodríguez-Roca et al. [65]. However, the combination of diuretics and ARBs was more common in our study (27.9% vs. 13.4%) [65]. Given that RAAS blockers are the only drugs that have proven effective in reducing the risk of end-stage renal disease [57], the different proportion of patients with CKD (21.7% in our study vs. 15.8% reported by Rodriguez-Roca et al.) [65] could partially explain this difference.

In our cohort, diuretics were widely used among older adults with HT. Practice guidelines [57] recommend diuretics, either alone or in combination with ACE inhibitors or ARBs, as a first-line option in the oldest age group to achieve a SBP target of 130–139 mmHg. However, their predominance over other agents warrants attention. This prescribing pattern may be explained by the coexistence of conditions such as HF, CKD, and volume overload, where diuretics are commonly indicated. Nevertheless, their use in frail older patients carries risks, including electrolyte disturbances, impaired renal function, and increased susceptibility to falls. Future work should therefore assess whether diuretic use is always clinically justified and reinforce the importance of individualized treatment and careful monitoring in this population.

Another relevant finding is that 25.9% of patients received three or more antihypertensive agents, reflecting the high burden of multimorbidity and treatment complexity in this population. While such prescribing patterns may be clinically justified, they also raise concerns about polypharmacy, which is strongly associated with adverse drug events. In this context, our findings emphasize the need to include deprescribing in routine care for frail older adults, ensuring that treatment decisions balance potential benefits with possible harms.

The percentage of patients treated with statins in our study reached 47.8%, which was slightly lower than that reported in the PICAF study (52.3%) [45]. This difference, along with the statistically significant sex differences found in our study (42.2% of women vs. 47.3% of men; *p* < 0.001), which were not observed in the PICAF study, may be explained by the prevalence of DLD in both studies (56.4% of men and 59.6% of women; *p* = 0.109 vs. 47.4% and 56.2%, respectively, in our study; *p* < 0.001). The finding that a substantial proportion of statin users had no previous CVD is consistent with the guidelines of the ESC [62] and the ACC [66], which recommend statin therapy for primary prevention in patients at high cardiovascular risk (e.g., diabetes, hypertension, elevated LDL-cholesterol, or high risk scores). This pattern reflects the application of preventive strategies in routine practice and illustrates how guideline recommendations are translated into real-world prescribing.

However, when these findings are interpreted in light of the 2019 meta-analysis by the Cholesterol Treatment Trialists’ Collaboration [67]—which reported proportional reductions in major vascular events across age groups for secondary prevention, but more heterogeneous and attenuated effects in primary prevention, particularly at older ages—the pattern observed in our study warrants a more nuanced interpretation. Although 60% of individuals receiving statins had no prior history of CVD, additional stratified analyses showed that statin use in this group was largely concentrated among patients with major cardiometabolic conditions, such as T2DM, DLD, and CKD, for whom statin-based primary-prevention is common. Moreover, statin use declined markedly with advancing age, suggesting a risk- and age-sensitive prescribing pattern rather than indiscriminate use in primary prevention.

The sex differences observed in cardiovascular conditions and medication use in this cohort are likely multifactorial [68]. Biological factors, including sex-related differences in cardiovascular pathophysiology and aging processes, may partly explain variations in disease patterns [69]. In parallel, social and behavioural determinants, such as lifetime exposure to smoking and alcohol, differences in social roles, and accumulated health disadvantages, may also contribute. Finally, differences in clinical practice patterns cannot be excluded, as previous studies have described lower intensity of preventive cardiovascular treatments in older women than men [70]. Given the descriptive design of this study, these findings should be interpreted as hypothesis-generating, and further research is needed to disentangle the relative contribution of biological, social, and healthcare-related factors.

Furthermore, the changes observed during the retrospective baseline period represent descriptive comparisons of yearly mean measurements between the earliest and latest available year within each subgroup, based on routinely collected clinical data. These should not be interpreted as modelled within-person trajectories or longitudinal trends.

From a clinical perspective, the treatment patterns observed in the Aged-Madrid cohort appear broadly aligned with current European guidelines for cardiovascular risk management in older adults [66], particularly regarding the widespread use of antihypertensive [57], lipid-lowering [55], and glucose-lowering therapies in patients with established cardiometabolic conditions. Nevertheless, given the descriptive design of this analysis and the lack of information on treatment intensity, dosing, adherence, and individual contraindications, these findings should be interpreted as reflecting general prescribing patterns rather than patient-level treatment appropriateness or achievement of guideline-recommended targets.

The high prevalence of multimorbidity and long-term pharmacological treatment observed in older adults in our cohort underscores the clinical relevance of deprescribing and treatment de-intensification as part of comprehensive cardiovascular risk management [71]. Although our study was not designed to identify overtreatment or to assess individual eligibility for deprescribing, these findings highlight the importance of regularly re-evaluating therapeutic intensity in the context of frailty, life expectancy, and competing risks, in line with recommendations from geriatric and cardiovascular societies [66,72].

The inclusion of an area-based deprivation index adds an equity-oriented perspective to the interpretation of cardiovascular risk in later life. Socioeconomic disadvantage is a well-established determinant of cardiovascular morbidity, and access to preventive care, and mortality, and its distribution within this cohort reinforces the need to consider social context when interpreting treatment patterns and planning risk reduction strategies [73,74]. Although we did not directly assess differences in treatment by socioeconomic status, the distribution of socioeconomic deprivation within the cohort, together with the high burden of cardiometabolic conditions and widespread use of preventive pharmacological therapies, supports the relevance of integrating social determinants of health into clinical and public health approaches aimed at improving cardiovascular outcomes among older populations.

### 4.1. Strengths

Our study provides a comprehensive representation of patients aged 75 years and older in the region of Madrid (Spain). It includes a 5-year retrospective baseline period (2015–2019) to establish trend changes in the collected variables. In addition to data being drawn from various sources, including EMRs from primary care, the present study contains data on the routine follow-up of elderly patients, which have been validated for several diseases. This will enable future studies based-on real-world data from 2020 to 2025 to assess the incidence of metabolic or cardiovascular events and mortality patterns in this cohort.

### 4.2. Weaknesses

The Aged-Madrid data were collected under real-world clinical conditions from EMRs, which limited access to certain variables (e.g., Barthel Index, frailty measures) and did not involve active search for symptoms or signs of any condition. Missing values were present in several clinical variables, which is less frequently recorded in routine practice. Reporting the extent of missing data provides a realistic characterization of the cohort, while future model development will require detailed assessment of missing data patterns and appropriate imputation methods.

We chose not to apply imputation methods, given the descriptive nature of the study. Our approach was to report percentages based on available data, acknowledging that missingness may reflect differences in age, sex, or care intensity. However, future explanatory and predictive modelling will require a detailed assessment of missing data patterns and the use of imputation strategies.

Another limitation is the absence of information on the duration of residence in the Madrid region. The cohort includes all individuals aged ≥75 years who were alive and publicly insured on 1 January 2020. However, we cannot distinguish long-term residents from those who moved to Madrid shortly before the index date. This may influence the completeness of historical clinical information and could reflect differences in cumulative exposures, healthcare use, or lifestyle patterns. Although all participants were receiving care under the same National Health System at cohort entry, this limitation should be considered when interpreting baseline characteristics and generalizing findings to populations with differences in residential stability.

A further limitation relates to medication-related information. Although pharmacy records enabled us to describe prescribed and dispensed treatments, data on medication adherence, treatment persistence, and individual clinical contraindications were not available. Consequently, the reported medication patterns reflect prescribing practices rather than confirmed long-term treatment exposure. Factors such as intolerance, frailty, drug–drug interactions, and patient preferences could not be assessed and should be considered when interpreting differences in medication use across subgroups.

Furthermore, information on dose, adherence, and treatment intensity was not available on our dataset. Therefore, our interpretation of treatment patterns refers only to the choice of drug classes (e.g., metformin as first-line therapy, diuretics as common antihypertensives) and not to dose or adherence. This limitation has been acknowledged to avoid overinterpretation of concordance with guidelines.

## 5. Conclusions

In adults aged 75 years and older, sex differences were evident across lifestyle factors, clinical conditions, biochemical and anthropometric parameters, and medication use. Men showed a higher prevalence of CVD and several cardiometabolic risk factors, including T2DM, AF, and prior AMI, stroke, and PAD. In contrast, females exhibited less favourable lipid profiles and lower use of lipid-lowering and antidiabetic therapies. Blood pressure levels and e-GFR were broadly comparable between sexes. Diuretics were the most frequently prescribed antihypertensive agents, reflecting age-related blood pressure patterns and the high burden of comorbidity. Although many statin users had no prior CVD, statin use in primary prevention was concentrated mainly among individuals with major cardiometabolic risk conditions and declined with advancing age. Taken together, these results are consistent with previous studies highlighting persistent sex-related gradients in cardiometabolic health, while also underscoring the complexity of cardiovascular risk management in very old adults and the need for individualized, context-aware strategies in routine clinical practice.

## Figures and Tables

**Table 1:** Baseline clinical characteristics of the Aged-Madrid cohort.

**A—Overall and By sex**							
				**By sex**			
		**Overall**		**Women**		**Men**	
**Number of participants**		587,603 (100.0%)		362,760/587,603 (61.7%)		224,843/587,603 (38.3%)	
**Age (years) ^a^**		83.5 (5.8)		84.0 (6.0)		82.7 (5.4)	
**Habits**	Alcohol consumption (Yes) ^a^	6680/587,603 (1.1%)		945/362,760 (0.3%)		5735/224,843 (2.6%)	
	Smoking (Yes) ^a^	28,497/587,603 (4.8%)		9745/362,760 (2.7%)		18,752/224,843 (8.3%)	
**Body mass index category**	Overweight	189,440/423,021 (44.8%)		106,145/259,350 (40.9%)		83,295/163,671 (50.9%)	
	Obesity	132,593/423,021 (31.3%)		87,506/259,350 (33.7%)		45,087/163,671 (27.5%)	
**Type 2 diabetes mellitus ^a^**		139,582/587,603 (23.8%)		78,100/362,760 (21.5%)		61,482/224,843 (27.3%)	
**Dyslipidaemia ^a^**		310,486/587,603 (52.8%)		203,902/362,760 (56.2%)		106,584/224,843 (47.4%)	
**Hypertension ^a^**		361,836/587,603 (61.6%)		235,856/362,760 (65.0%)		125,980/224,843 (56.0%)	
**Chronic kidney disease ^a^**		83,596/385,062 (21.7%)		53,966/241,264 (22.4%)		29,630/143,798 (20.6%)	
**Atrial fibrillation ^a^**		88,991/587,603 (15.1%)		50,078/362,760 (13.8%)		38,913/224,843 (17.3%)	
**Heart failure ^a^**		36,869/587,603 (6.3%)		23,689/362,760 (6.5%)		13,180/224,843 (5.9%)	
**Stroke ^a^**		35,649/587,603 (6.1%)		19,257/362,760 (5.3%)		16,392/224,843 (7.3%)	
**Acute myocardial infarction ^a^**		26,844/587,603 (4.6%)		8475/362,760 (2.3%)		18,369/224,843 (8.2%)	
**B—By age group**							
		**75–80 years old**	**81–85 years old**		**86–90 years old**		**>90 years old**
**Number of participants**		227,187/587,603 (38.7%)	158,891/587,603 (27.0%)		124,669/587,603 (21.2%)		76,856 S/587,603 (13.1%)
**Age (years) ^a^**		77.8 (1.5)	83.1 (1.4)		87.8 (1.4)		93.9 (2.9)
**Habits**	Alcohol consumption (Yes) ^a^	3633/227,187 (1.6%)	1873/158,891 (1.2%)		920/124,669 (0.7%)		254/76,856 (0.3%)
	Smoking (Yes) ^a^	16,856/227,187 (7.4%)	7468/158,891 (4.7%)		3284/124,669 (2.6%)		889/76,856 (1.2%)
**Body mass index category**	Overweight	73,435/163,735 (44.8%)	54,046/119,750 (45.1%)		41,534/92,367 (45.0%)		20,425/47,169 (43.3%)
	Obesity	55,955/163,735 (34.2%)	39,015/119,750 (32.6%)		27,023/92,367 (29.3%)		10,600/47,169 (22.5%)
**Type 2 diabetes mellitus ^a^**		52,813/227,187 (23.2%)	39,754/158,891 (25.0%)		31,136/124,669 (25.0%)		15,879/76,856 (20.7%)
**Dyslipidaemia ^a^**		124,662/227,187 (54.9%)	86,794/158,891 (54.6%)		65,288/124,669 (52.4%)		33,742/76,856 (43.9%)
**Hypertension ^a^**		130,286/227,187 (57.3%)	100,272/158,891 (63.1%)		81,747/124,669 (65.6%)		49,531/76,856 (64.4%)
**Chronic kidney disease ^a^**		17,644/149,542 (11.8%)	21,738/105,486 (20.6%)		24,521/82,511 (29.7%)		19,693/47,523 (41.4%)
**Atrial fibrillation ^a^**		23,372/227,187 (10.3%)	24,608/158,891 (15.5%)		25,027/124,669 (20.1%)		15,984/76,856 (20.8%)
**Heart failure ^a^**		6626/227,187 (2.9%)	9222/158,891 (5.8%)		11,583/124,669 (9.3%)		9438/76,856 (12.3%)
**Stroke ^a^**		9874/227,187 (4.3%)	9765/158,891 (6.1%)		9622/124,669 (7.7%)		6388/76,856 (8.3%)
**Acute myocardial infarction ^a^**		9238/227,187 (4.1%)	7535/158,891 (4.7%)		6329/124,669 (5.1%)		3742/76,856 (4.9%)

Age is expressed as the mean (standard deviation). Categorical variables are expressed as n/N (%), with N being the total number of participants with available information. For hypothesis testing by sex, the *t* test was applied, whereas analysis by age group was performed via one-way ANOVA. χ^2^ tests were used to compare categorical variables. All comparisons by sex and by age group were significant (*p* < 0.001). ^a^ No participants with missing values.

**Table 2:** Baseline analytical and blood pressure levels by sex and age group.

**A—Treated Patients**										
			**By sex**			**By age group**				
		**Overall**	**Women**	**Men**	**SMD**	**75–80 Years Old**	**81–85 Years Old**	**86–90 Years Old**	**>90 Years Old**	**SMD**
**For type 2 diabetes**	Fasting plasma glucose (mg/dL)	131.9 (30)	131.9 (30.4)	132.0 (28.9)	0.0	132.9 (29.2)	132.4 (29.7)	131.0 (29.8)	128.2 (30.7)	0.1
	HbA1c (%) *	7.0 (1.0)	7.0 (1.0)	7.0 (0.9)	0.1	7.0 (1.0)	7.0 (1.0)	7.0 (1.0)	7.0 (1.0)	0.0
**For dyslipidaemia**	Total cholesterol (mg/dL)	181.0 (31.5)	188.7 (29.9)	167.81 (29.79)	0.7	183.4 (31.27)	180.6 (31.3)	178.2 (31.7)	176.8 (32.4)	0.1
	LDL-cholesterol (mg/dL)	101.6 (26.6)	105.5 (26.2)	94.8 (25.7)	0.4	103.8 (26.47)	101.0 (26.3)	99.1 (26.5)	97.9 (27.2	0.1
	HDL-cholesterol (mg/dL)	55.5 (13.9)	58.9 (13.8)	49.6 (11.9)	0.7	55.4 (13.81)	55.7 (13.9)	55.5 (13.9)	55.1 (14.2)	0.0
	Triglycerides (mg/dL)	126.5 (53.9)	128.5 (53.6)	123.0 (54.4)	0.1	127.9 (54.84)	126.1 (53.8)	125.1 (52.9)	124.1 (51.9)	0.0
**For hypertension**	Systolic blood pressure (mmHg)	134.0 (11.0)	134.3 (10.9)	133.5 (11.2)	0.1	134.1 (10.9)	134.2 (10.9)	133.9 (11.7)	133.4 (11.6)	0.0
	Diastolic blood pressure (mmHg)	73.7 (6.6)	73.9 (6.5)	73.4 (6.8)	0.1	75.2 (6.6)	73.7 (6.4)	72.6 (6.4)	71.6 (6.5)	0.3
**B—Non-treated patients**										
			**By sex**			**By age group**				
		**Overall**	**Women**	**Men**	**SMD**	**75–80 years old**	**81–85 years old**	**86–90 years old**	**>90 years old**	**SMD**
**For type 2 diabetes**	Fasting plasma glucose (mg/dL)	115.4 (25)	114.3 (24.6)	117.3 (24.3)	0.1	118.2 (25.7)	116.6 (24.1)	114.2 (23.1)	111.6 (24.7)	0.2
	HbA1c (%)	6.4 (0.8)	6.4 (0.8)	6.4 (0.8)	0.0	6.4 (0.8)	6.4 (0.8)	6.4 (0.8)	6.4 (0.8)	0.0
**For dyslipidaemia**	Total cholesterol (mg/dL)	203.1 (32.8)	208.0 (31.7)	190.8 (32.6)	0.5	209.6 (30.7)	204.0 (32.1)	199.1 (33.4)	194.4 (34.3)	0.3
	LDL-cholesterol (mg/dL)	123.0 (27.7)	125.2 (27.1	117.3 (28.4)	0.3	127.9 (26.4)	123.4 (27.2)	119.7 (28.0)	117.0 (28.9)	0.2
	HDL-cholesterol (mg/dL)	57.5 (14.9)	60.0 (14.9)	50.9 (12.6)	0.7	58.8 (14.9)	58.0 (14.8)	56.9 (14.8)	54.9 (14.5)	0.1
	Triglycerides (mg/dL)	120.2 (49.9)	120.3 (48.5)	120.2 (53.4)	0.0	120.7 (51.3)	119.6 (49.7)	119.7 (48.4)	120.9 (49.4)	0.0
**For hypertension**	Systolic blood pressure (mmHg)	132.5 (12.6)	132.6 (12.7)	132.2 (12.5)	0.0	133.9 (12.5)	132.9 (12.5)	131.7 (12.5)	130.3 (12.8)	0.2
	Diastolic blood pressure (mmHg)	73.6 (7.4)	73.5 (7.4)	73.5 (7.4)	0.0	75.7 (7.5)	73.9 (7.2)	72.5 (7.1)	71.1 (7.1)	0.4

Data are expressed as mean (standard deviation). For hypothesis testing by sex, the *t* test was applied, and for each age group, ANOVA was used. * All comparisons by sex and age group were significant (*p* ≤ 0.05) except for HbA1c in treated patients by sex (*p* = 0.7), HbA1c in treated patients by age group (*p* = 0.1), and triglycerides in non-treated patients by sex (*p* = 0.8). Missing values—Fasting plasma glucose in treated patients: 5701/111,081 (5.1%); fasting plasma glucose in non-treated patients, 3889/28,501 (13.7%); total cholesterol in treated patients, 8588/210,397 (4.1%); total cholesterol in non-treated patients, 9651/100,089 (9.6%); LDL cholesterol in treated patients, 13,939/210,397 (6.6%); LDL cholesterol in non-treated patients, 12,248/100,089 (12.2%); HDL cholesterol in treated patients, 13,786/210,397 (6.6%); HDL cholesterol in non-treated patients, 12,142/100,089 (12.1%); triglycerides in treated patients, 10,432/210,397 (5.0%); triglycerides in non-treated patients, 10,626/100,089 (10.6%); systolic or diastolic blood pressure in treated patients, 14,894/315,558 (4.7%); systolic or diastolic blood pressure in non-treated patients, 10,870/45,558 (23.9%). SMD: standard mean difference, SMD by age group calculated for 75–80 years old vs. >90 years old.

**Table 3:** Baseline treatments for T2DM, HT and DLD in the Aged-Madrid cohort.

**A—Overall and By sex**							
			**By sex**				
	**Overall**		**Women**		**Men**		**V Cramer**
**Oral antidiabetics**	155,091/587,603 (26.4%)		82,951/362,760 (22.9%)		72,140/224,843 (32.1%)		0.1
Metformin	94,529/587,603 (16.1%)		50,624/362,760 (14.0%)		43,905/224,843 (19.5%)		0.1
DPP4 inhibitors	51,635/587,603 (8.8%)		28,175/362,760 (7.8%)		23,460/224,843 (10.4%)		0.1
**Insulin**	30,683/587,603 (5.2%)		17,842/362,760 (4.9%)		12,841/224,843 (5.7%)		0.0
**Diuretics**	316.698/587,603 (53.9%)		210.110/362,760 (57.9%)		106.588/224,843 (47.4%)		0.1
Loop diuretics	93,133/587,603 (15.8%)		60,779/362,760 (16.8%)		32,354/224,843 (14.4%)		0.0
Other diuretics	223,565/587,603 (38.0%)		149,331/362,760 (41.2%) *		74,234/224,843 (33.0%) *		0.1
**RAAS blockers**	309,576/587,603 (52.7%)		190,244/362,760 (52.6%)		119,332/224,843 (53.1%)		0.0
ACE inhibitors	161,018/587,603 (27.4%)		93,402/362,760 (25.7%)		67,616/224,843 (30.1%)		0.1
ARB	148,558/587,603 (25.3%)		96,842/362,760 (26.7%)		51,716/224,843 (23.0%)		0.0
**Calcium antagonists**	126,976/587,603 (21.6%)		77,328/362,760 (21.3%)		49,648/224,843 (22.1%)		0.0
**Beta-blockers**	117,694/587,603 (20.0%)		68,918/362,760 (19.0%)		48,776/224,843 (21.7%)		0.0
**Lipid-lowering agents**	280,825/587,603 (47.8%)		164,119/362,760 (45.2%)		116,706/224,843 (51.9%)		0.0
Statins	259,432/587,603 (44.2%)		152,995/362,760 (42.2%)		106,437/224,843 (47.3%)		0.0
Ezetimibe	21,393/587,603 (3.6%)		11,124/362,760 (3.1%)		10,269/224,843 (4.6%)		0.0
**B—By age group**							
	**75–80 years old**	**81–85 years old**		**86–90 years old**		**>90 years old**	**V Cramer**
**Oral antidiabetics**	66,591/227,187 (29.3%)	45,114/158,891 (28.4%)		31,018/124,669 (24.9%)		12,368/76,856 (16.1%)	0.1
Metformin	40,800/227,187 (18.0%)	27,553/158,891 (17.3%)		18,728/124,669 (15.0%)		7448/76,856 (9.7%)	0.1
DPP4 inhibitors	20,238/227,187 (8.9%)	15,341/158,891 (9.7%)		11,344/124,669 (9.1%)		4712/76,856 (6.1%)	0.0
**Insulin**	11,586/227,187 (5.1%)	8992/158,891 (5.7%)		6975/124,669 (5.6%)		3130/76,856 (4.1%)	0.0
**Diuretics**	94.067/227,187 (41.4%)	86.851/158,891 (54.7%)		81.402/124,669 (65.3%)		54,378/76,856 (70.8%)	0.1
Loop diuretics	19,633/227,187 (8.6%)	24,008/158,891 (15.1%)		27,769/124,669 (22.3%)		21,723/76,856 (28.3%)	0.2
Other diuretics	74,434/227,187 (32.8%)	62,843/158,891 (39.6%)		53,633/124,669 (43.0%)		32,655/76,856 (42.5%)	0.1
**RAAS blockers**	117,238/227,187 (51.6%)	88,643/158,891 (55.8%)		68,159/124,669 (54.7%)		35,536/76,856 (46.2%)	0.1
ACE inhibitors	60,514/227,187 (26.6%)	45,554/158,891 (28.7%)		35,733/124,669 (28.7%)		19,217/76,856 (25.0%)	0.0
ARB	56,724/227,187 (25.0%)	43,089/158,891 (27.1%)		32,426/124,669 (26.0%)		16,319/76,856 (21.2%)	0.0
**Calcium antagonists**	45,674/227,187 (20.1%)	36,317/158,891 (22.9%)		29,180/124,669 (23.4%)		15,805/76,856 (20.6%)	0.0
**Beta-blockers**	42,743/227,187 (18.8%)	33,627/158,891 (21.2%)		27,271/124,669 (21.9%)		14,053/76,856 (18.3%)	0.0
**Lipid-lowering agents**	119,064/227,187 (52.4%)	82,360/158,891 (51.8%)		57,028/124,669 (45.7%)		22,373/76,856 (29.1%)	0.1
Statins	108,290/227,187 (47.7%)	76,036/158,891 (47.9%)		53,591/124,669 (43.0%)		21,515/76,856 (28.0%)	0.1
Ezetimibe	10,774/227,187 (4.7%)	6324/158,891 (4.0%)		3437/124,669 (2.8%)		858/76,856 (1.1%)	0.1

Data are expressed as n/N (%) for categorical variables, with N as the total number of participants with available information. χ^2^ tests were used to compare categorical variables. * All comparisons by sex were significant (*p* < 0.001 except for other diuretics, *p* = 0.05). All comparisons by age group were significant (*p* < 0.001). Cramer’s V was interpreted as strong (> 0.5), moderate (0.3–0.5), or weak (<0.3). ACE inhibitors: angiotensin-converting enzyme inhibitors; ARB: angiotensin II receptor blockers; DPP4 inhibitors: dipeptidyl peptidase-4 inhibitors; RAAS blockers: renin–angiotensin–aldosterone system blockers.

**Table 4:** Use of statins in patients without cardiovascular disease in the Aged-Madrid cohort.

**A—Overall and By sex**							
				**By sex**			
		**Overall**		**Women**		**Men**	
**Type 2 diabetes**	Non-treated with statins	34,706/78,944 (44.0%)		20,821/48,538 (42.9%)		13,885/30,406 (45.7%)	
	Treated with statins	44,238/78,944 (56.0%)		27,717/48,538 (57.1%)		16,521/30,406 (54.3%)	
**Dyslipidaemia**	Non-treated with statins	78,958/201,685 (39.1%)		57,359/142,820 (40.2%)		21,599/58,865 (36.7%)	
	Treated with statins	122,727/201,685 (60.9%)		85,461/142,820 (59.8%)		37,266/58,865 (63.3%)	
**Hypertension**	Non-treated with statins	135,202/236,168 (57.2%)		93,077/163,860 (56.8%)		42,125/72,308 (58.3%)	
	Treated with statins	100,966/236,168 (42.8%)		70,783/163,860 (43.2%)		30,183/72,308 (41.7%)	
**Chronic kidney disease**	Non-treated with statins	24,486/44,065 (55.6%)		17,120/30,919 (55.4%)		7366/13,146 (56.0%)	
	Treated with statins	19,579/44,065 (44.4%)		13,799/30,919 (44.6%)		5780/13,146 (44.0%)	
**B—By age group**							
		**By age group**					
		**75–80 years old**	**81–85 years old**		**86–90 years old**		**>90 years old**
**Type 2 diabetes**	Non-treated with statins	12,598/33,537 (37.6%)	9203/22,334 (41.2%)		7754/15,439 (50.2%)		5151/7634 (67.5%)
	Treated with statins	20,939/33,537 (62.4%)	13,131/22,334 (58.8%)		7685/15,439 (49.8%)		2483/7634 (32.5%)
**Dyslipidaemia**	Non-treated with statins	31,949/90,914 (35.1%)	20,361/55,874 (36.4%)		16,262/37,301 (43.6%)		10,386/17,596 (59.0%)
	Treated with statins	58,965/90,914 (64.9%)	35,513/55,874.0 (63.6%)		21,039/37,301 (56.4%)		7210/17,596 (41.0%)
**Hypertension**	Non-treated with statins	49,493/95,189 (52.0%)	35,610/65,549 (54.3%)		29,379/48,060 (61.1%)		20,720/27,370 (75.7%)
	Treated with statins	45,696/95,189 (48.0%)	29,939/65,549 (45.7%)		18,681/48,060 (38.9%)		6650/27,370 (24.3%)
**Chronic kidney disease**	Non-treated with statins	4804/10,625 (45.2%)	5745/11,809 (48.6%)		6928/12,177 (56.9%)		7009/9454 (74.1%)
	Treated with statins	5821/10,625 (54.8%)	6064/11,809 (51.4%)		5249/12,177 (43.1%)		2445/9454 (25.9%)

Data are expressed as n/N (%) for categorical variables, with N as the total number of participants with available information. For comparisons of categorical variables, the χ^2^ test was used. All comparisons by sex and by age group were significant (*p* < 0.001). No missing values were observed for the variables included in this table.

## Data Availability

The datasets generated and/or analysed during the current study are available from the corresponding author upon reasonable request.

## Supplementary Materials

The following supporting information can be downloaded at: https://www.mdpi.com/article/10.3390/jcm15020571/s1, Table S1: Distribution of missing values across clinical variables; Table S2: Other baseline characteristics and comorbidities of the Aged-Madrid cohort by sex and by age-group; Table S3: Baseline lifestyle factors, body mass index categories, and cardiometabolic and cardiovascular comorbidities of the Aged-Madrid cohort stratified by socioeconomic deprivation status; Table S4: Other baseline analytical parameters associated with cardiovascular risk of the Aged-Madrid cohort by sex and by age-group; Table S5: Other baseline treatments of the Aged-Madrid cohort by sex and by age-group; Table S6: Variation in Body Mass Index within the global population, by sex and by age group, during the pre-observational period (2015–2019); Table S7: Variation in FPG within the global population, by sex and by age group, during the pre-observational period (2015–2019); Table S8: Variation in LDL-cholesterol within the global population, by sex and by age group, during the pre-observational period (2015–2019); Table S9: Variation in SBP within the global population, by sex and by age group, during the pre-observational period (2015–2019); Table S10: Variation in DBP within the global population, by sex and by age group, during the pre-observational period (2015–2019); Figure S1: Presence and absence of data across study variables; Figure S2: Missing data patterns.

## List of Abbreviations

ACE	Angiotensin-converting enzyme
AF	Atrial fibrillation
ARBs	Angiotensin II receptor blockers
AMI	Acute myocardial infarction
BMI	Body mass index
CCDE	Composite cardiovascular disease endpoints
CKD	Chronic kidney disease
CKD-EPI	Chronic Kidney Disease Epidemiology Collaboration
CMBD	Hospital discharge data
CVD	Cardiovascular disease
CVRCs	Cardiovascular risk comorbidities
DBP	Diastolic blood pressure
DLD	Dyslipidaemia
DPP-4	dipeptidyl peptidase-4
e-GFR	Glomerular filtration rate
EMRs	Electronic medical records
EU	European Union
FPG	Fasting plasma glucose
GLP-1 RA	GLP-1 receptor agonists
HT	Hypertension
INDEF	National Death Index of the Ministry of Health
INE	National Statistics Institute
PAD	Peripheral arterial disease
RAAS	Renin‒angiotensin‒aldosterone system
SBP	Systolic blood pressure
SD	Standard deviation
SGLT2	Sodium-Glucose Co-Transporter 2
SMDs	Standard mean differences
T2DM	Type 2 diabetes mellitus
