# Supplementary material for: Cohort Profile: A Descriptive Analysis of Patients Aged 75 Years and Older with Public Health Coverage in Madrid at Baseline, Including a 5-Year Preobservational Period (2015–2019)
Source: J Clin Med. 2026 Jan 10;15(2):571. doi: 10.3390/jcm15020571 (PMC12841762; doi:10.3390/jcm15020571)
Supplement: Supplementary file 1 [file jcm-15-00571-s001.zip › jcm-4027974-supplementary.pdf]

Supplementary Table S1. Distribution of missing values across clinical variables

A-Overview

Variable	Label	Missing values (%)
f_barthel	Barthel index	65.81%
fpg_15	Average fasting plasma glucose in 2015	42.80%
fpg_16	Average fasting plasma glucose in 2016	42.80%
fpg_17	Average fasting plasma glucose in 2017	42.50%
fpg_18	Average fasting plasma glucose in 2018	41.33%
fpg_19	Average fasting plasma glucose in 2019	40.66%
f_erc	Chronic kidney disease diagnosis	34.50%
ckdepi_prom_15_19	Average CKDEPI 2015-2019	34.50%
f_imc_3	BMI diagnosis	28.01%
imc_num	Number of BMI measurements between 2015–2019	28.01%
imc_prim_med	First BMI measurement between 2015 and 2019	28.01%
imc_ult_med	Last BMI measurement between 2015 and 2019	28.01%
LDL_prom	Average plasma LDL-cholesterol 2015-2019	15.44%
LDL_num	Number of plasma LDL-cholesterol measurements between 2015–2019	15.44%
HDL_prom	Average plasma HDL-cholesterol 2015-2019	15.40%
HDL_num	Number of plasma HDL-cholesterol measurements between 2015–2019	15.40%
gpa_prom_15_19	Average fasting plasma glucose 2015-2019	14.60%
gpa_num	Number of plasma fasting plasma glucose measurements between 2015–2019	14.60%
gpa_prim_med	First plasma fasting plasma glucose measurement between 2015 and 2019	14.60%
gpa_ult_med	Last plasma fasting plasma glucose measurement between 2015 and 2019	14.60%
trig_prom	Average plasma triglycerides 2015-2019	13.26%
trig_num	Number of plasma triglycerides measurements between 2015–2019	13.26%
pas_prom	Average systolic blood pressure 2015-2019	13.20%
pas_prim_med	First systolic blood pressure measurement between 2015 and 2019	13.20%

pas_ult_med	Last systolic blood pressure measurement between 2015 and 2019	13.20%
pad_prom	Average diastolic blood pressure 2015-2019	13.20%
pad_prim_med	First diastolic blood pressure measurement between 2015 and 2019	13.20%
pad_ult_med	Last diastolic blood pressure measurement between 2015 and 2019	13.20%
f_pre_t2dm	Pre-type 2 diabetes diagnosis	12.30%
tot_col_num	Number of plasma total-cholesterol measurements between 2015–2019	12.11%
tot_col_prom	Average plasma total-cholesterol 2015-2019	12.11%
indpriv	Deprivation index	0.04%

Aged-Madrid variables whose percentage of missing values exceeds 0.00%

B-Stratified by age-group

variable	75-80 years old	81-85 years old	>=86 years-old
HDL_num	28,093 (15.46%)	24,630 (14.22%)	37,516 (16.12%)
HDL_prom	28,093 (15.46%)	24,630 (14.22%)	37,516 (16.12%)
LDL_num	28,221 (15.53%)	24,810 (14.32%)	37,703 (16.2%)
LDL_prom	28,221 (15.53%)	24,810 (14.32%)	37,703 (16.2%)
ckdepi_prom_15_19	62,416 (34.36%)	58,022 (33.5%)	82,103 (35.28%)
f_barthel	153,228 (84.34%)	119,342 (68.9%)	114,157 (49.06%)
f_erc	62,416 (34.36%)	58,022 (33.5%)	82,103 (35.28%)
f_imc_3	52,050 (28.65%)	42,923 (24.78%)	69,609 (29.91%)
f_pre_t2dm	25,413 (13.99%)	21,040 (12.15%)	29,509 (12.68%)
fpg_15	78,604 (43.27%)	72,179 (41.67%)	100,647 (43.25%)
fpg_16	78,376 (43.14%)	72,026 (41.58%)	100,812 (43.32%)
fpg_17	77,486 (42.65%)	71,904 (41.51%)	100,304 (43.1%)
fpg_18	75,425 (41.52%)	69,667 (40.22%)	97,802 (42.03%)
fpg_19	74,351 (40.93%)	68,794 (39.72%)	95,746 (41.14%)
gpa_num	28,507 (15.69%)	23,849 (13.77%)	33,376 (14.34%)
gpa_prim_med	28,507 (15.69%)	23,849 (13.77%)	33,376 (14.34%)
gpa_prom_15_19	28,507 (15.69%)	23,849 (13.77%)	33,376 (14.34%)
gpa_ult_med	28,507 (15.69%)	23,849 (13.77%)	33,376 (14.34%)
imc_num	52,050 (28.65%)	42,923 (24.78%)	69,609 (29.91%)
imc_prim_med	52,050 (28.65%)	42,923 (24.78%)	69,609 (29.91%)
imc_ult_med	52,050 (28.65%)	42,923 (24.78%)	69,609 (29.91%)
indpriv	97 (0.05%)	89 (0.05%)	69 (0.03%)
pad_prim_med	26,447 (14.56%)	20,074 (11.59%)	30,927 (13.29%)
pad_prom	26,447 (14.56%)	20,074 (11.59%)	30,927 (13.29%)
pad_ult_med	26,447 (14.56%)	20,074 (11.59%)	30,927 (13.29%)
pas_prim_med	26,447 (14.56%)	20,074 (11.59%)	30,927 (13.29%)
pas_prom	26,447 (14.56%)	20,074 (11.59%)	30,927 (13.29%)
pas_ult_med	26,447 (14.56%)	20,074 (11.59%)	30,927 (13.29%)
tot_col_num	22,995 (12.66%)	19,414 (11.21%)	28,784 (12.37%)
tot_col_prom	22,995 (12.66%)	19,414 (11.21%)	28,784 (12.37%)
trig_num	25,219 (13.88%)	21,380 (12.34%)	31,305 (13.45%)
trig_prom	25,219 (13.88%)	21,380 (12.34%)	31,305 (13.45%)
N	181,676 (30.92%)	173,216 (29.48%)	232,711 (39.6%)

B-Stratified by sex

variable	**Women**	**Men**
HDL_num	52,354 (14.43%)	37,885 (16.85%)
HDL_prom	52,354 (14.43%)	37,885 (16.85%)
LDL_num	52,678 (14.52%)	38,056 (16.93%)
LDL_prom	52,678 (14.52%)	38,056 (16.93%)
ckdepi_prom_15_19	121,496 (33.49%)	81,045 (36.05%)
f_barthel	224,037 (61.76%)	162,690 (72.36%)
f_erc	121,496 (33.49%)	81,045 (36.05%)
f_imc_3	103,410 (28.51%)	61,172 (27.21%)
f_pre_t2dm	44,855 (12.36%)	31,107 (13.83%)
fpg_15	149,621 (41.25%)	101,809 (45.28%)
fpg_16	149,773 (41.29%)	101,441 (45.12%)
fpg_17	148,713 (40.99%)	100,981 (44.91%)
fpg_18	144,715 (39.89%)	98,179 (43.67%)
fpg_19	142,142 (39.18%)	96,749 (43.03%)
gpa_num	49,784 (13.72%)	35,948 (15.99%)
gpa_prim_med	49,784 (13.72%)	35,948 (15.99%)
gpa_prom_15_19	49,784 (13.72%)	35,948 (15.99%)
gpa_ult_med	49,784 (13.72%)	35,948 (15.99%)
imc_num	103,410 (28.51%)	61,172 (27.21%)
imc_prim_med	103,410 (28.51%)	61,172 (27.21%)
imc_ult_med	103,410 (28.51%)	61,172 (27.21%)
indpriv	123 (0.03%)	132 (0.06%)
pad_prim_med	47,403 (13.07%)	30,045 (13.36%)
pad_prom	47,403 (13.07%)	30,045 (13.36%)
pad_ult_med	47,403 (13.07%)	30,045 (13.36%)
pas_prim_med	47,403 (13.07%)	30,045 (13.36%)
pas_prom	47,403 (13.07%)	30,045 (13.36%)
pas_ult_med	47,403 (13.07%)	30,045 (13.36%)
tot_col_num	41,809 (11.53%)	29,384 (13.07%)
tot_col_prom	41,809 (11.53%)	29,384 (13.07%)
trig_num	45,537 (12.55%)	32,367 (14.4%)
trig_prom	45,537 (12.55%)	32,367 (14.4%)
N	362,760 (61.74%)	224,843 (38.26%)

D-Stratified by sex and age-group

variable	Women Age=75-80	Women Age=81-85	Women Age=>=86	Men Age=75-80	Men Age=81-85	Men Age=>=86
**HDL_num**	14,698 (14.31%)	13,616 (13.1%)	24,040 (15.4%)	13,395 (16.96%)	11,014 (15.9%)	13,476 (17.59%)
**HDL_prom**	14,698 (14.31%)	13,616 (13.1%)	24,040 (15.4%)	13,395 (16.96%)	11,014 (15.9%)	13,476 (17.59%)
**LDL_num**	14,748 (14.36%)	13,746 (13.22%)	24,184 (15.49%)	13,473 (17.06%)	11,064 (15.97%)	13,519 (17.65%)
**LDL_prom**	14,748 (14.36%)	13,746 (13.22%)	24,184 (15.49%)	13,473 (17.06%)	11,064 (15.97%)	13,519 (17.65%)
**ckdepi_prom_15_19**	33,924 (33.03%)	33,432 (32.16%)	54,140 (34.68%)	28,492 (36.08%)	24,590 (35.5%)	27,963 (36.5%)
**f_barthel**	84,637 (82.4%)	67,852 (65.28%)	71,548 (45.83%)	68,591 (86.86%)	51,490 (74.33%)	42,609 (55.62%)
**f_erc**	33,924 (33.03%)	33,432 (32.16%)	54,140 (34.68%)	28,492 (36.08%)	24,590 (35.5%)	27,963 (36.5%)
**f_imc_3**	28,933 (28.17%)	25,625 (24.65%)	48,852 (31.29%)	23,117 (29.27%)	17,298 (24.97%)	20,757 (27.1%)
**f_pre_t2dm**	13,831 (13.47%)	11,960 (11.51%)	19,064 (12.21%)	11,582 (14.67%)	9,080 (13.11%)	10,445 (13.64%)
**fpg_15**	42,989 (41.85%)	41,294 (39.73%)	65,338 (41.85%)	35,615 (45.1%)	30,885 (44.58%)	35,309 (46.09%)
**fpg_16**	42,836 (41.71%)	41,349 (39.78%)	65,588 (42.01%)	35,540 (45.01%)	30,677 (44.28%)	35,224 (45.98%)
**fpg_17**	42,154 (41.04%)	41,300 (39.73%)	65,259 (41.8%)	35,332 (44.74%)	30,604 (44.18%)	35,045 (45.75%)
**fpg_18**	40,960 (39.88%)	39,995 (38.48%)	63,760 (40.84%)	34,465 (43.65%)	29,672 (42.83%)	34,042 (44.44%)
**fpg_19**	40,288 (39.22%)	39,371 (37.88%)	62,483 (40.03%)	34,063 (43.14%)	29,423 (42.47%)	33,263 (43.42%)
**gpa_num**	15,102 (14.7%)	13,291 (12.79%)	21,391 (13.7%)	13,405 (16.98%)	10,558 (15.24%)	11,985 (15.65%)
**gpa_prim_med**	15,102 (14.7%)	13,291 (12.79%)	21,391 (13.7%)	13,405 (16.98%)	10,558 (15.24%)	11,985 (15.65%)
**gpa_prom_15_19**	15,102 (14.7%)	13,291 (12.79%)	21,391 (13.7%)	13,405 (16.98%)	10,558 (15.24%)	11,985 (15.65%)
**gpa_ult_med**	15,102 (14.7%)	13,291 (12.79%)	21,391 (13.7%)	13,405 (16.98%)	10,558 (15.24%)	11,985 (15.65%)
**imc_num**	28,933 (28.17%)	25,625 (24.65%)	48,852 (31.29%)	23,117 (29.27%)	17,298 (24.97%)	20,757 (27.1%)
**imc_prim_med**	28,933 (28.17%)	25,625 (24.65%)	48,852 (31.29%)	23,117 (29.27%)	17,298 (24.97%)	20,757 (27.1%)
**imc_ult_med**	28,933 (28.17%)	25,625 (24.65%)	48,852 (31.29%)	23,117 (29.27%)	17,298 (24.97%)	20,757 (27.1%)
**indpriv**	37 (0.04%)	43 (0.04%)	43 (0.03%)	60 (0.08%)	46 (0.07%)	26 (0.03%)
**pad_prim_med**	14,408 (14.03%)	11,707 (11.26%)	21,288 (13.64%)	12,039 (15.25%)	8,367 (12.08%)	9,639 (12.58%)
**pad_prom**	14,408 (14.03%)	11,707 (11.26%)	21,288 (13.64%)	12,039 (15.25%)	8,367 (12.08%)	9,639 (12.58%)
**pad_ult_med**	14,408 (14.03%)	11,707 (11.26%)	21,288 (13.64%)	12,039 (15.25%)	8,367 (12.08%)	9,639 (12.58%)
**pas_prim_med**	14,408 (14.03%)	11,707 (11.26%)	21,288 (13.64%)	12,039 (15.25%)	8,367 (12.08%)	9,639 (12.58%)
**pas_prom**	14,408 (14.03%)	11,707 (11.26%)	21,288 (13.64%)	12,039 (15.25%)	8,367 (12.08%)	9,639 (12.58%)
**pas_ult_med**	14,408 (14.03%)	11,707 (11.26%)	21,288 (13.64%)	12,039 (15.25%)	8,367 (12.08%)	9,639 (12.58%)
**tot_col_num**	12,288 (11.96%)	10,902 (10.49%)	18,619 (11.93%)	10,707 (13.56%)	8,512 (12.29%)	10,165 (13.27%)
**tot_col_prom**	12,288 (11.96%)	10,902 (10.49%)	18,619 (11.93%)	10,707 (13.56%)	8,512 (12.29%)	10,165 (13.27%)
**trig_num**	13,364 (13.01%)	11,935 (11.48%)	20,238 (12.96%)	11,855 (15.01%)	9,445 (13.63%)	11,067 (14.45%)
**trig_prom**	13,364 (13.01%)	11,935 (11.48%)	20,238 (12.96%)	11,855 (15.01%)	9,445 (13.63%)	11,067 (14.45%)
**N**	102,711 (17.48%)	103,940 (17.69%)	156,109 (26.57%)	78,965 (13.44%)	69,276 (11.79%)	76,602 (13.04%)

Supplementary Figure S1. Presence and absence of data across study variables

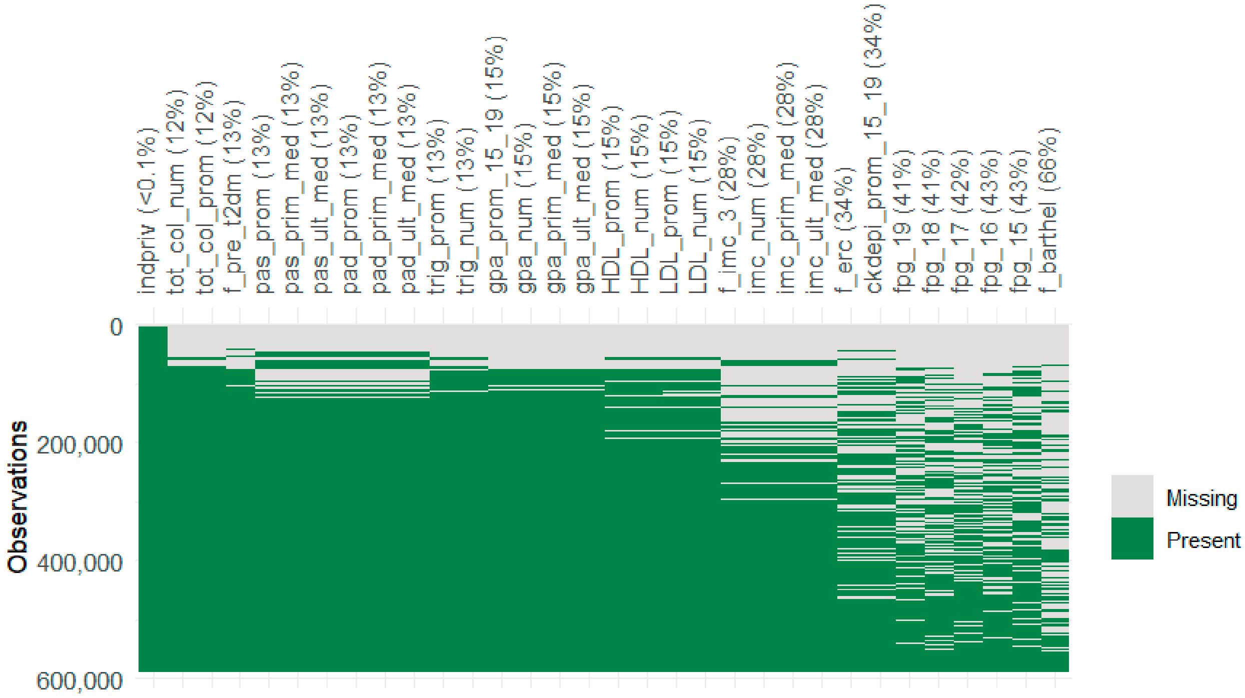

Heatmap showing the presence (green) and absence (grey) of data across the selected clinical variables and all observations. Each row represents an individual record and each column corresponds to a variable, with the percentage of missing values per variable indicated at the top. Structured patterns of missingness are observed across variables and observations, suggesting that missing data are not completely random but tend to cluster in specific subsets of variables and records, likely related to the joint availability of certain clinical tests or records.

Supplementary Figure S2. Missing data patterns

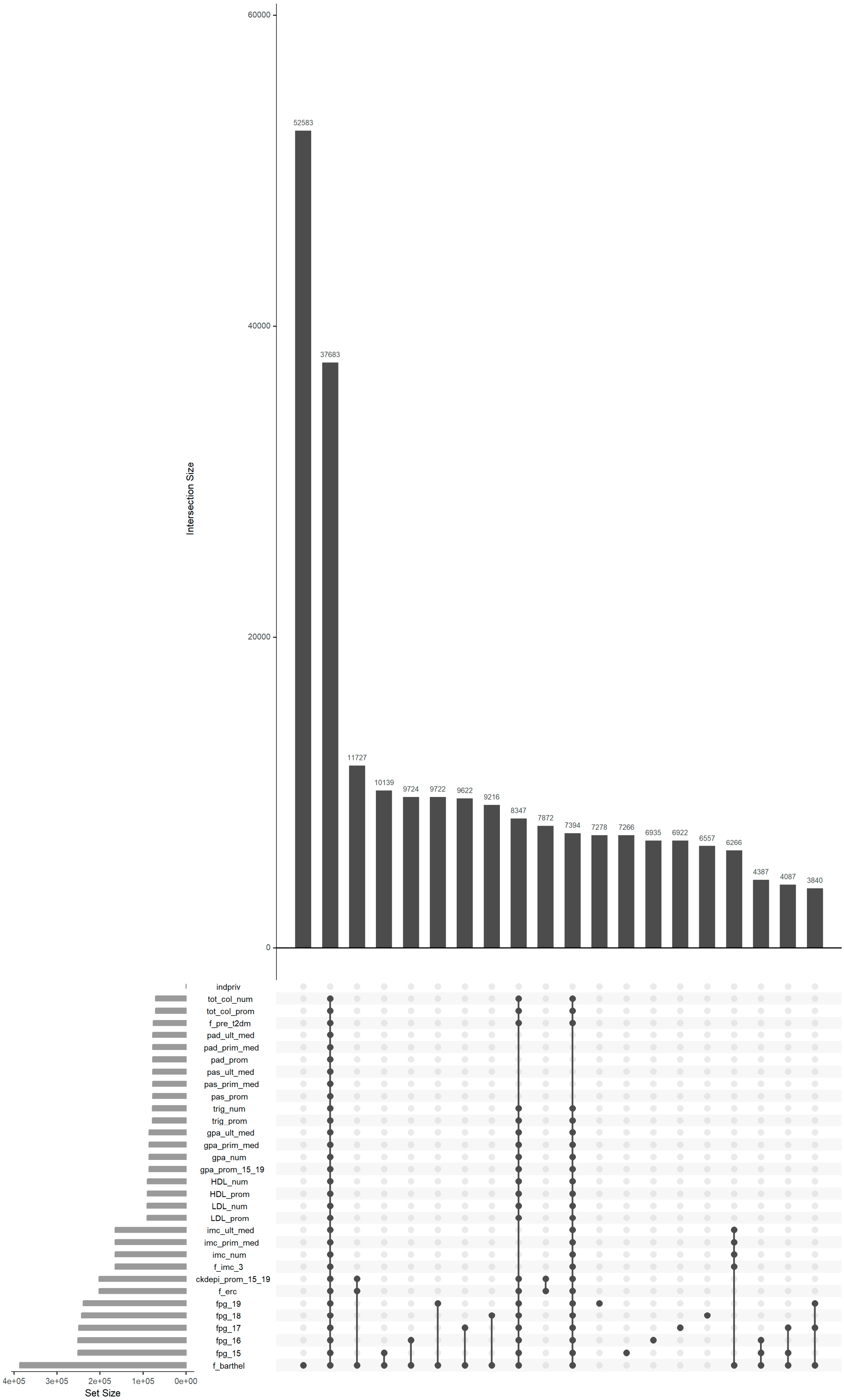

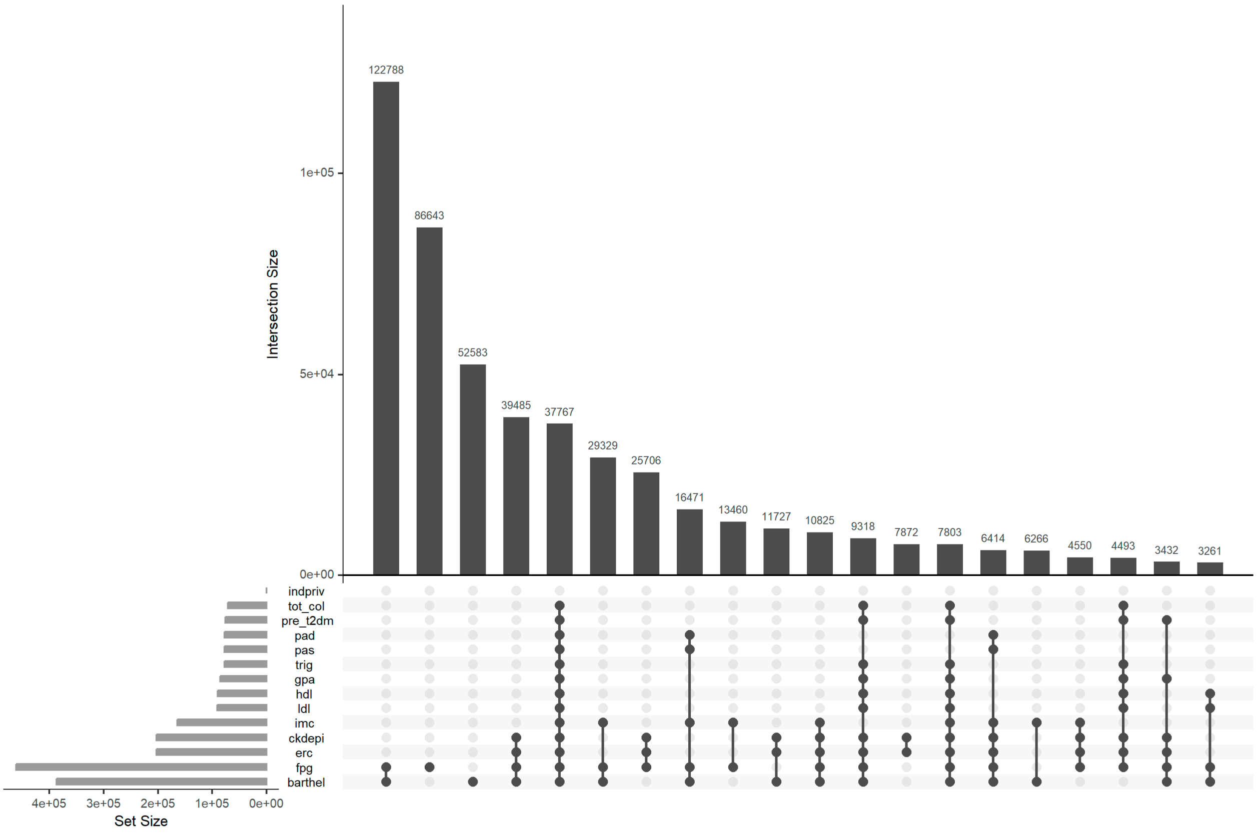

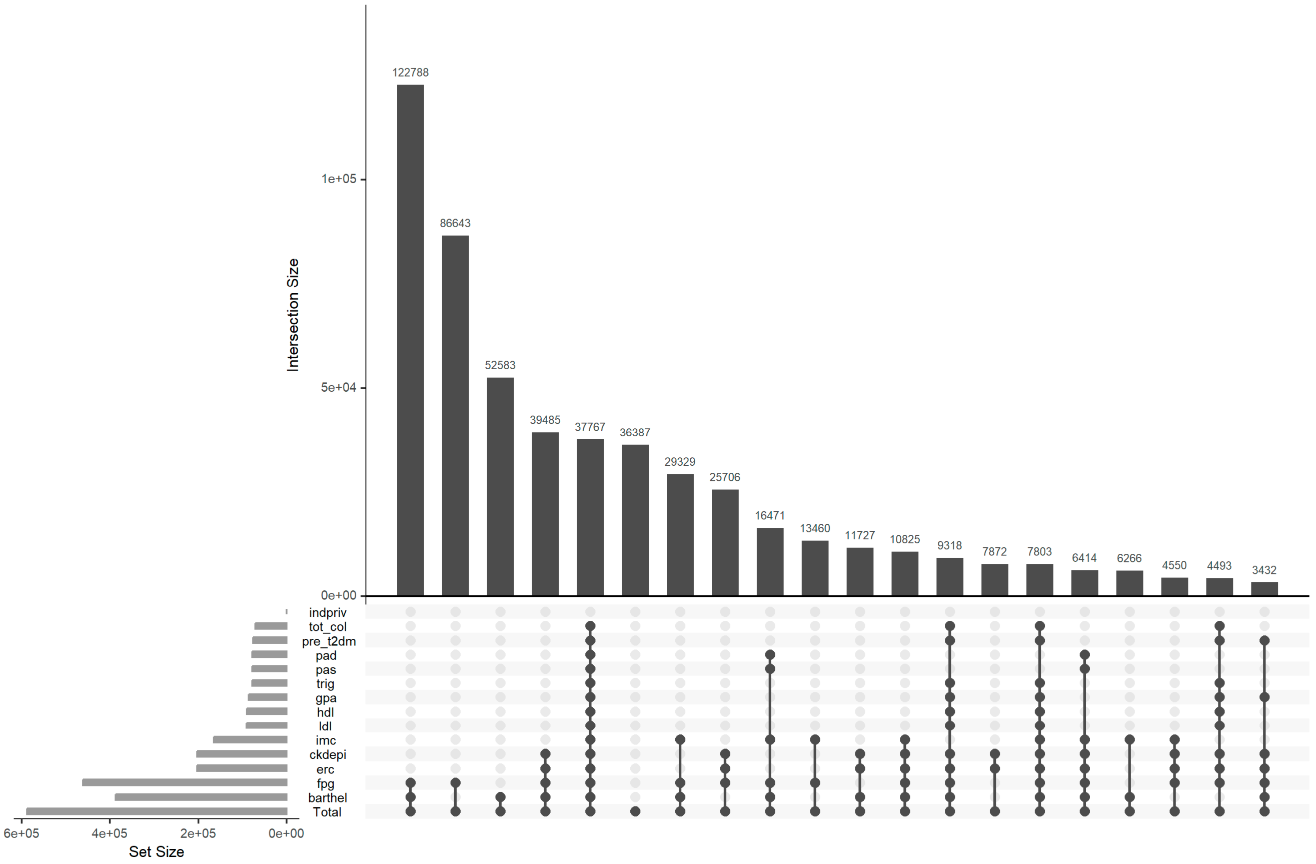

UpSet plot of missing data patterns in the selected clinical variables. The horizontal bars on the left represent the total number of records with missing values per variable, while the vertical bars indicate the size of the intersections between specific combinations of variables with missing data. Connected dots show the co-occurrence of missingness across variables, with

several dominant patterns observed. These results suggest that missingness is not completely random, but rather tends to cluster in specific combinations of variables, likely associated with the joint collection of certain clinical tests or records.

Supplementary Table S2. Other baseline characteristics and comorbidities of the Aged-Madrid cohort by sex and by age-group

		By sex		By age-group			
	**Overall**	**Women**	**Men**	**75-80 years old**	**81-85 years old**	**86-90 years old**	**> 90 years old**
**Pre- Type 2 Diabetes Mellitus [a]**	76,216 / 511,641 (14.9%)	44,073 / 317,905 (13.9%)	32,143 / 193,736 (16.6%)	30,282 / 196,101 (15.4%)	21,582 / 139,874 (15.4%)	16,250 / 110,498 (14.7%)	8,102 / 65,168 (12.4%)
**Transient ischemic attack**	27,393 / 587,603 (4.7%)	16,298 / 362,760 (4.5%)	11,095 / 224,843 (4.9%)	7,670 / 227,187 (3.4%)	7,221 / 158,891 (4.5%)	7,127 / 124,669 (5.7%)	5,375 / 76,856 (7.0%)
**Angina pectoris**	26,808 / 587,603 (4.6%)	11,848 / 362,760 (3.3%)	14,960 / 224,843 (6.7%)	8,869 / 227,187 (3.9%)	7,597 / 158,891 (4.8%)	6,544 / 124,669 (5.2%)	3,798 / 76,856 (4.9%)
**Peripheral arterial disease**	25,483 / 587,603 (4.3%)	8,701 / 362,760 (2.4%)	16,782 / 224,843 (7.5%)	8,969 / 227,187 (3.9%)	7,434 / 158,891 (4.7%)	5,953 / 124,669 (4.8%)	3,127 / 76,856 (4.1%)
**CCDE3**	80,409 / 587,603 (13.7%)	34,528 / 362,760 (9.5%)	45,881 / 224,843 (20.4%)	25,705 / 227,187 (11.3%)	22,468 / 158,891 (14.1%)	20,010 / 124,669 (16.1%)	12,226 / 76,856 (15.9%)
**CCDE4**	108,069 / 587,603 (18.4%)	53,785 / 362,760 (14.8%)	54,284 / 224,843 (24.1%)	30,532 / 227,187 (13.4%)	29,282 / 158,891 (18.4%)	28,722 / 124,669 (23.0%)	19,533 / 76,856 (25.4%)
**Skin Cancer**	38,406 / 587,603 (6.5%)	20,186 / 362,760 (5.6%)	18,220 / 224,843 (8.1%)	11,611 / 227,187 (5.1%)	10,523 / 158,891 (6.6%)	9,546 / 124,669 (7.7%)	6,726 / 76,856 (8.8%)
**Prostate cancer**	19,732 / 587,603 (3.4%)	0 / 362,760 (0.0%)	19,732 / 224,843 (8.8%)	8,113 / 227,187 (3.6%)	5,789 / 158,891 (3.6%)	4,132 / 124,669 (3.3%)	1,707 / 76,856 (2.2%)
**Colorectal cancer**	18,497 / 587,603 (3.1%)	8,729 / 362,760 (2.4%)	9,768 / 224,843 (4.3%)	6,494 / 227,187 (2.9%)	5,306 / 158,891 (3.3%)	4,426 / 124,669 (3.6%)	2,271 / 76,856 (3.0%)
**Breast cancer**	13,174 / 587,603 (2.2%)	13,161 / 362,760 (3.6%)	13 / 224,843 (0.0%)	5,305 / 227,187 (2.3%)	3,612 / 158,891 (2.3%)	2,690 / 124,669 (2.2%)	1,567 / 76,856 (2.0%)
**Bladder cancer**	9,694 / 587,603 (1.6%)	1,869 / 362,760 (0.5%)	7,825 / 224,843 (3.5%)	3,755 / 227,187 (1.7%)	2,796 / 158,891 (1.8%)	2,140 / 124,669 (1.7%)	1,003 / 76,856 (1.3%)
**Trachea, bronchus and lung cancer**	3,459 / 587,603 (0.6%)	959 / 362,760 (0.3%)	2,500 / 224,843 (1.1%)	1,649 / 227,187 (0.7%)	1,052 / 158,891 (0.7%)	582 / 124,669 (0.5%)	176 / 76,856 (0.2%)
**Alzheimer disease**	46,273 / 587,603 (7.9%)	33,101 / 362,760 (9.1%)	13,172 / 224,843 (5.9%)	7,549 / 227,187 (3.3%)	11,509 / 158,891 (7.2%)	15,079 / 124,669 (12.1%)	12,136 / 76,856 (15.8%)
**Barthel Index Score**							
No or light dependence	165,619 / 200,876 (82.4%)	112,923 / 138,723 (81.4%)	52,696 / 62,153 (84.8%)	34,928.0 / 39,667.0 (88.1%)	47,960.0 / 55,348.0 (86.7%)	50,315.0 / 61,034.0 (82.4%)	32,416.0 / 44,827.0 (72.3%)
Moderate dependence	18,327/ 200,876 (9.1%)	13,625.0 / 138,723.0 (9.8%)	4,702 / 62,153 (7.6%)	2,239.0 / 39,667.0 (5.6%)	3,683.0 / 55,348.0 (6.7%)	5,797.0 / 61,034.0 (9.5%)	6,608.0 / 44,827.0 (14.7%)
Severe or total dependence	16,930 / 200,876 (8.4%)	12,175.0 / 138,723.0 (8.8%)	4,755 / 62,153 (7.7%)	2,500.0 / 39,667.0 (6.3%)	3,705.0 / 55,348.0 (6.7%)	4,922.0 / 61,034.0 (8.1%)	5,803.0 / 44,827.0 (12.9%)

Data are expressed as mean (standard deviation) for numeric variables or as n/N (%) for categorical variables with N as total number of participants with available information; For hypothesis testing by sex, the t test was applied, whereas analysis by age group was performed via the one-way analysis of means (anova) test. χ2 tests were used to compare categorical variables. *All comparisons by sex and by age-group were significant (p-value < 0.001).

a According to IDF criteria, Pre- Type 2 Diabetes Mellitus was defined as FPG $\geq$ 100 mg/dL - < 126 mg/dL among patients diagnosed as non-diabetic type 2 diabetics or who are not being treated with antidiabetics

CCDE: Composite cardiovascular disease endpoints; **CCDE3:** Acute myocardial infarction, stroke, and peripheral arterial disease**; CCDE4:** CCDE3 and Heart failure; **FPG**: Fasting plasma glucose; **T2DM**: Type 2 diabetes

Supplementary Table S3. Baseline lifestyle factors, body mass index categories, and cardiometabolic and cardiovascular comorbidities of the Aged-Madrid cohort stratified by socioeconomic deprivation status

**Characteristic**	**Less disadvantaged** N = 392,437[1]	**More disadvantaged** N = 194,911[1]	**p-value**[2]
**Age**	83.6 (5.9)	83.2 (5.6)	<0.001
**Alcohol consumption**	4,062 / 392,437 (1.0%)	2,618 / 194,911 (1.3%)	<0.001
**Tobacco consumption**	18,820 / 392,437 (4.8%)	9,677 / 194,911 (5.0%)	0.005
**BMI category**			<0.001
Under/Normal weight	70,922 / 271,176 (26.2%)	30,066 / 151,842 (19.8%)	
Overweight	121,531 / 271,176 (44.8%)	67,907 / 151,842 (44.7%)	
Obesity	78,723 / 271,176 (29.0%)	53,869 / 151,842 (35.5%)	
**Type 2 DM**	86,834 / 392,437 (22.1%)	52,747 / 194,911 (27.1%)	<0.001
**Dyslipidaemia**	202,627 / 392,437 (51.6%)	107,857 / 194,911 (55.3%)	<0.001
**Hypertension**	236,255 / 392,437 (60.2%)	125,578 / 194,911 (64.4%)	<0.001
**Chronic kidney disease**	48,479 / 241,766 (20.1%)	35,116 / 143,292 (24.5%)	<0.001
**Atrial fibrillation**	59,055 / 392,437 (15.0%)	29,936 / 194,911 (15.4%)	0.002
**Heart failure**	23,549 / 392,437 (6.0%)	13,319 / 194,911 (6.8%)	<0.001
**Stroke**	23,374 / 392,437 (6.0%)	12,275 / 194,911 (6.3%)	<0.001
**Acute myocardial infarction**	17,331 / 392,437 (4.4%)	9,513 / 194,911 (4.9%)	<0.001

Data are expressed as n/N (%) for categorical variables with N as total number of participants with available information. $\chi 2$ tests were used to compare categorical variables.

Supplementary Table S4. Other baseline analytical parameters associated with cardiovascular risk of the Aged-Madrid cohort by sex and by age-group

		By sex		By age-group			
	**Overall**	**Women**	**Men**	**75-80 years old**	**81-85 years old**	**86-90 years old**	**> 90 years old**
**Albuminuria (g/dL)**	4.17 (0.53)	4.13 (0.55)	4.20 (0.52)	4.26 (0.46)	4.20 (0.50)	4.10 (0.53)	3.90 (0.61)
Unknown	268,944	161,493	107,451	109,498	74,150	54,297	30,999
**Albuminuria/creatinine (mg/g)**	11 (24)	11 (21)	12 (25)	9 (18)	11 (23)	13 (24)	17 (28)
Unknown	317,955	201,321	116,634	122,781	82,236	65,946	46,992
**Microalbuminuria (µg/mL)**	9 (14)	8 (11)	10 (20)	8 (11)	9 (14)	10 (18)	11 (21)
Unknown	400,557	256,366	144,191	155,351	106,160	83,593	55,453
**Creatinine (mg/dL)**	0.85 (0.30)	0.78 (0.23)	0.98 (0.28)	0.82 (0.26)	0.85 (0.29)	0.88 (0.33)	0.91 (0.37)
Unknown	74,176	43,458	30,718	29,970	18,464	13,939	11,803

Data are expressed as mean (standard deviation) for numeric variables or as n/N (%) for categorical variables with N as total number of participants with available information; For hypothesis testing by sex, the t test was applied, whereas analysis by age group was performed via the one-way analysis of means (anova) test. χ^2 tests were used to compare categorical variables. *All comparisons by sex and by age-group were significant (p-value < 0.001).

Supplementary Table S5. Other baseline treatments of the Aged-Madrid cohort by sex and by age-group

		By sex		By age-group			
	**Overall**	**Women**	**Men**	**75-80 years old**	**81-85 years old**	**86-90 years old**	**> 90 years old**
**SGLT2 inhibitors**	8,565/587,603 (1.5%)	3,947/362,760 (1.1%)	4,618/224,843 (2.1%)	5,368/227,187 (2.4%)	2,121/158,891 (1.3%)	886/124,669 (0.7%)	190/76,856 (0.2%)
**GLP1 RA**	2,787 / 587,603 (0.5%)	1,611 / 362,760 (0.4%)	1,176 / 224,843 (0.5%)	1,987 / 227,187 (0.9%)	620 / 158,891 (0.4%)	172 / 124,669 (0.1%)	8 / 76,856 (0.0%)
**Thiazolidinediones**	362/587,603 (0.1%)	205/362,760 (0.1%)*	157/224,843 (0.1%)*	185/227,187 (0.1%)	99/158,891 (0.1%)	60/124,669 (0.0%)	18/76,856 (0.0%)
**Anticoagulants**	100,909 / 587,603 (17.2%)	58,012 / 362,760 (16.0%)	42,897 / 224,843 (19.1%)	28,912 / 227,187 (12.7%)	28,777 / 158,891 (18.1%)	27,450 / 124,669 (22.0%)	15,770 / 76,856 (20.5%)
**Antiplatelets**	141,367 / 587,603 (24.1%)	74,460 / 362,760 (20.5%)	66,907 / 224,843 (29.8%)	48,053 / 227,187 (21.2%)	39,320 / 158,891 (24.7%)	33,297 / 124,669 (26.7%)	20,697 / 76,856 (26.9%)
**Antialdosterone agents**	22,849 / 587,603 (3.9%)	13,617 / 362,760 (3.8%)	9,232 / 224,843 (4.1%)	6,701 / 227,187 (2.9%)	6,424 / 158,891 (4.0%)	6,038 / 124,669 (4.8%)	3,686 / 76,856 (4.8%)
**Alpha-blockers**	17,753 / 587,603 (3.0%)	8,022 / 362,760 (2.2%)	9,731 / 224,843 (4.3%)	6,822 / 227,187 (3.0%)	5,246 / 158,891 (3.3%)	3,952 / 124,669 (3.2%)	1,733 / 76,856 (2.3%)
**NSAIDs**	56,664 / 587,603 (9.6%)	39,299 / 362,760 (10.8%)	17,365 / 224,843 (7.7%)	29,906 / 227,187 (13.2%)	15,380 / 158,891 (9.7%)	8,321 / 124,669 (6.7%)	3,057 / 76,856 (4.0%)
**iCOX**	9,444 / 587,603 (1.6%)	7,159 / 362,760 (2.0%)	2,285 / 224,843 (1.0%)	4,733 / 227,187 (2.1%)	2,764 / 158,891 (1.7%)	1,492 / 124,669 (1.2%)	455 / 76,856 (0.6%)

Data are expressed as n/N (%) for categorical variables with N as total number of participants with available information; χ2 tests were used to compare categorical variables.

*All comparisons by sex were significant (p-value < 0.001 except for Thiazolidinediones $p < 0.05$), unless for Other diuretics ($p < 0.067$) and Resin ($p < 0.2$). All comparisons by age-group were significant (p-value < 0.001).

GLP1 RA: Glucagon-like peptide 1 receptor agonists; **iCOX:** Cyclooxygenase inhibitor**; NSAIDs:** Nonsteroidal anti-inflammatory drugs; **SGLT2 inhibitors**: Sodium-Glucose Co-Transporter 2 inhibitors;

Supplementary Table S6. Variation in Body Mass Index within the global population, by sex and by age group, during the pre-observational period (2015–2019).

	N	Number of BMI measurements [mean (SD)]	First BMI (Kg/m^2) measurement [mean (SD)]	Last BMI (Kg/m^2) measurement [mean (SD)]	Number of years between measurements [median (IQR)]	Variation of BMI (Kg/m^2)	p-value
**Overall**	346,284	11.4 (10.1)	28.7 (4.7)	28.1 (4.7)	3.9 (2.5)	-0.7 (2.2)	<0.001
**Sex**							
Female	212,707	11.4 (10.0)	28.9 (5.0)	28.2 (5.1)	3.9 (2.5)	-0.7 (2.3)	<0.001
Male	133,577	11.3 (10.1)	28.5 (4.0)	27.9 (4.1)	3.9 (2.5)	-0.6 (2.0)	<0.001
**Age-group**							
75-80 years old	131,928	11.2 (9.9)	29.1 (4.8)	28.7 (4.8)	3.9 (2.5)	-0.5 (2.1)	<0.001
81-85 years old	99,919	11.9 (10.2)	28.9 (4.6)	28.2 (4.7)	4.0 (2.3)	-0.6 (2.2)	<0.001
86-90 years old	77,192	11.6 (10.2	28.4 (4.5)	27.6 (4.6)	3.9 (2.4)	-0.9 (2.3)	<0.001
> 90 years old	37,245	10.3 (9.7)	27.5 (4.4)	26.5 (4.5)	3.4 (2.6)	-1.0 (2.4	<0.001

For patients with two measurements at least. For hypothesis testing of variation of BMI, the paired t test was applied. **BMI:** Body Mass Index

Supplementary Table S7. Variation in FPG within the global population, by sex and by age group, during the pre-observational period (2015–2019).

	N	Number of FPG measurements [mean (SD)]	First FPG (mg/dL) measurement [mean (SD)]	Last FPG (mg/dL) measurement [mean (SD)]	Number of years between measurements [median (IQR)]	Variation of FPG (mg/dL)	p-value
**Overall**	440,604	5.0 (2.5)	103.6 (29.1)	101.3 (28.2)	3.6 (1.6)	-2.3 (26.2	<0.001
**Sex**							
Female	277,910	5.1 (2.6)	101.7 (28.2)	99.7 (27.7)	3.6 (1.5)	-2.0 (25.5)	<0.001
Male	162,694	4.8 (2.5)	106.8 (30.3)	104.0 (28.6)	3.5 (1.7)	-2.8 (27.3)	<0.001
**Age-group**							
75-80 years old	167,919	4.9 (2.5)	104.0 (29.5)	102.2 (27.6)	3.6 (1.6)	-1.8 (25.2)	<0.001
81-85 years old	120,959	5.0 (2.6)	104.5 (29.6	102.5 (28.6	3.6 (1.6)	-2.0 (26.7)	<0.001
86-90 years old	95,756	5.0 (2.6)	103.6 (28.7)	101.0 (28.6)	3.6 (1.7)	-2.5 (26.8)	<0.001
> 90 years old	55,970	5.0 (2.6)	100.2 (27.6)	96.2 (27.5)	3.5 (1.7)	-3.9 (27.1)	<0.001

For patients with two measurements at least. For hypothesis testing of variation of FPG, the paired t test was applied. **FPG:** Fasting Plasma Glucose

Supplementary Table S8. Variation in LDL-cholesterol within the global population, by sex and by age group, during the pre-observational period (2015–2019).

	N	Number of LDL-cholesterol measurements [mean (SD)]	First LDL-cholesterol (mg/dL) measurement [mean (SD)]	Last LDL-cholesterol (mg/dL) measurement [mean (SD)]	Number of years between measurements [median (IQR)]	Variation of LDL-cholesterol (mg/dL)	p-value
**Overall**	428,045	4.7 (2.3)	109.4 (31.8)	102.3 (31.6)	102.3 (31.6)	-7.1 (29.7)	<0.001
**Sex**							
Female	270,107	4.8 (2.3)	113.0 (31.6)	106.7 (31.4)	3.7 (1.7)	-6.4 (30.7)	<0.001
Male	157,938	4.6 (2.2)	103.2 (31.1)	94.9 (30.5)	3.6 (1.8)	-8.3 (27.8)	<0.001
**Age-group**							
75-80 years old	166,236	4.8 (2.3)	112.2 (31.8)	104.6 (31.2)	3.8 (1.7)	-7.7 (29.6)	<0.001
81-85 years old	117,944	4.8 (2.3)	108.8 (31.5)	101.8 (31.4)	3.7 (1.8)	-7.1 (29.4)	<0.001
86-90 years old	91,817	4.7 (2.3)	106.8 (31.6)	100.2 (31.8)	3.7 (1.8)	-6.6 (29.8)	<0.001
> 90 years old	52,048	4.6 (2.3)	106.3 (31.7)	100.2 (32.1)	3.6 (1.9)	-6.0 (30.4)	<0.001

For patients with two measurements at least. For hypothesis testing of variation of LDL-cholesterol, the paired t test was applied

Supplementary Table S9. Variation in SBP within the global population, by sex and by age group, during the pre-observational period (2015–2019).

	N	Number of SBP measurements [mean (SD)]	First SBP (mmHg) measurement [mean (SD)]	Last SBP (mmHg) measurement [mean (SD)]	Number of years between measurements [median (IQR)]	Variation of SBP (mmHg)	p-value
**Overall**	462,725	15.0 (13.0)	133.0 (16.5)	131.8 (15.9)	4.3 (1.9)	-1.2 (19.8)	<0.001
**Sex**							
Female	287,088	15.2 (13.1)	133.3 (16.5)	132.3 (16.0	4.3 (1.9)	-1.0 (20.0)	<0.001
Male	175,637	14.5 (12.8)	132.6 (16.5	130.9 (15.7)	4.2 (2.0)	-1.6 (19.4)	<0.001
**Age-group**							
75-80 years old	174,033	13.9 (12.2)	132.9 (16.4)	131.9 (15.1)	4.2 (2.1)	-1.0 (19.0)	<0.001
81-85 years old	129,247	15.7 (13.2)	133.2 (16.5)	132.2 (15.8)	4.3 (1.8)	-1.0 (19.7)	<0.001
86-90 years old	102,138	16.1 (13.7)	133.1 (16.6)	131.7 (16.4)	4.3 (1.8)	-1.4 (20.4)	<0.001
> 90 years old	57,307	14.7 (13.3)	132.8 (17.0)	130.5 (17.3)	4.2 (2.0)	-2.4 (21.0)	<0.001

For patients with two measurements at least. For hypothesis testing of variation of SBP, the paired t test was applied. **SBP:** Systolic Blood Pressure

Supplementary Table S10. Variation in DBP within the global population, by sex and by age group, during the pre-observational period (2015–2019).

	N	Number of DBP measurements [mean (SD)]	First DBP (mmHg) measurement [mean (SD)]	Last DBP (mmHg) measurement [mean (SD)]	Number of years between measurements [median (IQR)]	Variation of DBP (mmHg)	p-value
**Overall**	462,725	15.0 (13.0)	74.0 (9.6)	72.4 (9.6)	4.3 (1.9)	-1.5 (11.6)	<0.001
**Sex**							
Female	287,088	15.2 (13.1)	74.2 (9.6)	72.7 (9.5)	4.3 (1.9)	-1.5 (11.6)	<0.001
Male	175,637	14.5 (12.8)	73.6 (9.6)	71.9 (9.5)	4.2 (2.0)	-1.7 (11.4	<0.001
**Age-group**							
75-80 years old	174,033	13.9 (12.2)	75.4 (9.6)	73.7 (9.3)	4.2 (2.1)	-1.7 (11.3)	<0.001
81-85 years old	129,247	15.7 (13.2)	73.9 (9.5)	72.4 (9.5)	4.3 (1.8)	-1.5 (11.5)	<0.001
86-90 years old	102,138	16.1 (13.7)	72.9 (9.4)	71.5 (9.6)	4.3 (1.8)	-1.4 (11.8)	<0.001
> 90 years old	57,307	14.7 (13.3)	71.8 (9.5)	70.4 (9.8)	4.2 (2.0)	-1.4 (12.0)	<0.001

For patients with two measurements at least. For hypothesis testing of variation of DBP, the paired t test was applied. **DBP:** Diastolic Blood Pressure
